# Genetic mapping, synteny, and physical location of two loci for *Fusarium oxysporum* f. sp. *tracheiphilum* race 4 resistance in cowpea [*Vignaunguiculata* (L.) Walp]

**DOI:** 10.1007/s11032-013-9991-0

**Published:** 2013-12-13

**Authors:** Marti O. Pottorff, Guojing Li, Jeffery D. Ehlers, Timothy J. Close, Philip A. Roberts

**Affiliations:** 1 Department of Botany and Plant Sciences, University of California Riverside, Riverside, CA USA; 2 Zhejiang Academy of Agricultural Sciences, Hangzhou, People’s Republic of China; 3 Bill and Melinda Gates Foundation, Seattle, WA USA; 4 Department of Nematology, University of California Riverside, Riverside, CA USA

**Keywords:** Cowpea, Fusarium wilt, Disease resistance, Candidate genes, Genomics, Synteny

## Abstract

Fusarium wilt is a vascular disease caused by the fungus *Fusarium* *oxysporum* f.sp. *tracheiphilum* (Fot) in cowpea [*Vigna* *unguiculata* (L.) Walp]. In this study, we mapped loci conferring resistance to Fot race 4 in three cowpea RIL populations: IT93K-503-1 × CB46, CB27 × 24-125B-1, and CB27 × IT82E-18/Big Buff. Two independent loci which confer resistance to Fot race 4 were identified, *Fot4*-*1* and *Fot4*-*2*. *Fot4*-*1* was identified in the IT93K-503-1 (resistant) × CB46 (susceptible) population and was positioned on the cowpea consensus genetic map, spanning 21.57–29.40 cM on linkage group 5. The *Fot4*-*2* locus was validated by identifying it in both the CB27 (resistant) × 24-125B-1 (susceptible) and CB27 (resistant) × IT82E-18/Big Buff (susceptible) populations. *Fot4*-*2* was positioned on the cowpea consensus genetic map on linkage group 3; the minimum distance spanned 71.52–71.75 cM whereas the maximum distance spanned 64.44–80.23 cM. These genomic locations of *Fot4*-*1* and *Fot4*-*2* on the cowpea consensus genetic map, relative to *Fot3*-*1* which was previously identified as the locus conferring resistance to Fot race 3, established that all three loci were independent. The *Fot4*-*1* and *Fot4*-*2* syntenic loci were examined in *Glycine max*, where several disease-resistance candidate genes were identified for both loci. In addition, *Fot4*-*1* and *Fot4*-*2* were coarsely positioned on the cowpea physical map. *Fot4*-*1* and *Fot4*-*2* will contribute to molecular marker development for future use in marker-assisted selection, thereby expediting introgression of Fot race 4 resistance into future cowpea cultivars.

**Electronic supplementary material:**

The online version of this article (doi:10.1007/s11032-013-9991-0) contains supplementary material, which is available to authorized users.

## Introduction

*Fusarium oxysporum* f. sp. *tracheiphilum* (Fot) is a soil-borne fungal pathogen that causes vascular wilt disease in cowpea [*Vigna* *unguiculata* (L.) Walp] (Armstrong and Armstrong 1981). The pathogen enters the plant through the root system and invades vascular tissue, causing wilting and leaf chlorosis and often stunting the entire plant. Broad irregular patches of affected plants are visible in infested cowpea fields. The external symptoms typically become evident during the flowering and early pod development stages resulting in high mortality in the affected areas with severe overall yield loss. Worldwide, the occurrence of Fusarium infecting cowpeas has been reported in the Northern Territory of Australia, northeastern parts of Brazil, and Nigeria (Summerell et al. 2011; Assunção et al. 2003; Armstrong and Armstrong 1980). Fusarium wilt of cowpea is a significant problem in the United States, especially in the southeastern states and California (Hare 1953).

In California, the prevalence of the disease stimulated breeding efforts to develop Fusarium resistance in cowpea from the 1930s onward (Patel 1985). In conjunction with the use of resistance in commercial cowpea cultivars, several races of Fot have evolved (races 1, 2, 3, and 4) which are identified according to differential interactions on cowpea genotypes with different resistance backgrounds (Hare 1953; Patel 1985; Smith et al. 1999). Fot race 3 has been the most prevalent and widespread race within the state of California (Smith et al. 1999) and several cultivars with resistance have been grown as a primary disease management tactic (Pottorff et al. 2012). However, in recent years, widely grown cowpea cultivars which were resistant to Fot race 3, such as California Blackeye 46, showed Fusarium disease symptoms in some fields, indicating that a new race had evolved which required a new focus in breeding for resistance (Davis and Frate 2007). Alternative disease management practices such as applications of fungicides are not feasible due to economic constraints as well as possible health and environmental concerns. Host resistance is therefore an effective and preferred solution for managing the disease in cowpea and new cultivars for production in the United States must have resistance to both Fot race 3 and race 4. Several new cultivars have been bred specifically to incorporate resistance to Fot race 4, including California Blackeye 27 (Ehlers et al. 2000) and the recently released California Blackeye 50 (Ehlers et al. 2009). These cultivars were developed using traditional breeding methods that involved screening and identifying appropriate resistant germplasm sources and then introgressing the resistance trait, often taking a decade or more to release a new cowpea cultivar. Precision breeding using marker-assisted selection with trait-linked markers could reduce the length of breeding time to less than half. However, the efficiency will depend on the extent of recombination between the trait determinant and marker based on the genetic distance between them. To improve breeding efficiency, gene-based ‘perfect markers’ could be developed through the identity of the genetic determinants for Fot race 4 resistance, as we reported recently for resistance to Fot race 3 in cowpea (Pottorff et al. 2012).

Molecular genetic tools and genomic resources have been developed for cowpea with an objective of enhancing breeding programs for the improvement of cowpea varieties for the United States, India, Brazil, and numerous countries in Africa and Asia. These genomic resources have been integrated by using a 1536-single nucleotide polymorphism (SNP) genotyping platform and include an expressed sequence tag (EST)-derived SNP cowpea consensus genetic map, known syntenic relationships between cowpea, *M. truncatula*, *G. max* and *A. thaliana*, and a cowpea EST sequence collection housed in HarvEST:Cowpea database (http://harvest.ucr.edu) (Muchero et al. 2009; Lucas et al. 2011). The cowpea physical map which has been anchored to the cowpea consensus genetic map using the same SNP genotyping platform is currently available (http://phymap.ucdavis.edu/cowpea). In addition, more than 500 diverse cowpea accessions have been SNP-genotyped and a first draft of the cowpea genome sequence has been assembled (www.harvest-blast.org). These resources will enable dissection of underlying genetic components of target agronomic traits using quantitative trait locus (QTL) analysis and association mapping. In this study, greenhouse inoculation experiments were used to identify QTLs conferring resistance against Fot race 4 in three cowpea recombinant inbred line (RIL) populations. Two loci which confer resistance to Fot race 4 were identified, *Fot4*-*1* and *Fot4*-*2*. The target outcome of this study will be to develop molecular markers closely linked to the *Fot4*-*1* and *Fot4*-*2* resistance genes for application in resistance breeding.

## Materials and methods

### Plant materials

Three cowpea RIL populations which segregate for Fot race 4 resistance were used for QTL mapping studies. The IT93K-503-1 (resistant) × CB46 (susceptible) population consisted of 113 lines advanced to the F10 generation using single seed descent. IT93K-503-1 is an advanced breeding line developed by the International Institute for Tropical Agriculture (IITA) with strong resistance to Fot race 4. CB46 was bred for resistance to Fot race 3 but is highly susceptible to Fot race 4 (Davis and Frate 2007).

The CB27 (resistant) × 24-125B-1 (susceptible) population consisted of 90 lines that were advanced to the F9 generation using single seed descent. CB27 was bred for resistance to several pathogens including root-knot nematodes and Fot race 4 and also for heat tolerance (Ehlers et al. 2000). 24-125B-1 is an advanced breeding line from the Institute of Agricultural Research for Development (IRAD) and is susceptible to Fot race 4 (Kitch et al. 2001).

The CB27 (resistant) × IT82E-18/Big Buff (susceptible) population consisted of 162 RILs and was advanced to the F8 generation by single seed descent. IT82E-18 is an advanced breeding line developed at IITA which was released as cultivar Big Buff in Australia (Imrie 1995). IT82E-18 is highly susceptible to Fot race 4. All cowpea RIL populations were obtained from the University of California Riverside cowpea germplasm collection.

### Inoculum preparation

Two strains of Fot race 4, which originated from infected cowpea plants in Bakersfield, California, were used for inoculation cultures. Individual isolates were developed from single spore lines. Isolates were dried and stored on sterile potato dextrose agar (PDA) plates at −80 °C. 1-cm^2^ plugs were cut from frozen Fusarium-containing PDA plates and transferred aseptically to flasks containing 500 ml of potato-dextrose broth, then incubated in a shaker at 27 °C, 30 rpm under lighted conditions for 3 days. The liquid culture was strained through four layers of cheesecloth to eliminate mycelia and the spore concentration was adjusted to 1.0 × 10^6^ microconidia per ml using a hemocytometer.

Plants were inoculated using a modified root-dip inoculation method described by Rigert and Foster (1987). Modifications to the root-dip method were as follows: 10 greenhouse-grown seeds per RIL were planted in seeding trays filled with vermiculite and watered daily for 1 week. After 1 week, five replicate seedlings per RIL were gently uprooted, the distal half of the root system was clipped and the remaining root system dipped for 1 min in suspended inoculum with a concentration of 1 × 10^6^ spores/ml. Inoculated seedlings were transplanted into 3.8-l pots and watered daily with greenhouse day temperatures set to 28 °C and night temperatures to 16 °C.

### Phenotyping

Plants were evaluated 35 days post-inoculation for Fusarium disease symptoms. The wilting/stunting phenotype was evaluated by approximating the percentage of wilting or stunting to the entire plant similar to the disease severity index (DSI) utilized by the Centro International de Agricultura Tropical (CIAT) (Pastor-Corrales and Abawi 1987; Fall et al. 2001). The reddish-brown vascular discoloration, which is the necrosis caused by the fungus as it moves both vertically and horizontally throughout the vascular system, was evaluated by uprooting the entire plant and slicing the stem vertically to evaluate the extent of the disease symptoms internally (Online Resource 1). The severity of the Fusarium symptoms was evaluated on a 0–5 rating scale for the wilting/stunting and vascular discoloration phenotypes. A score of 0 indicated a healthy plant with no signs of disease, 1 = approximately 10 % of the plant showing symptoms of disease, 2 = approximately 25 % of the plant showing symptoms of disease, 3 = approximately 50 % of the plant showing symptoms, 4 = approximately 75 % of the plant showing symptoms, and 5 = 100 % of the plant showing disease symptoms. Five replicates per RIL were evaluated individually then averaged to determine the disease severity for each RIL.

### Genetic maps

All populations and parental lines were genotyped at the F8 generation with bi-allelic SNP markers using the 1536 Illumina GoldenGate Assay previously described in Muchero et al. (2009).

A SNP-based genetic map for the IT93K-503-1 × CB46 population was developed previously and was included in the cowpea consensus genetic map (Lucas et al. 2011). The IT93K503-1 × CB46 genetic map consisted of eleven linkage groups, was approximately 734 cM length, and was generated using 113 RILs and 423 SNP markers (Lucas et al. 2011).

The SNP-based genetic map for the CB27 × 24-125B-1 population was also developed previously and was included in the cowpea consensus genetic map (Lucas et al. 2011). The CB27 × 24-125B-1 genetic map was generated using 339 SNP markers and 90 RILs, consisted of sixteen linkage groups, and was approximately 600 cM in length (Lucas et al. 2011).

The CB27 × IT82E-18/Big Buff genetic map was generated using 162 RILs and 419 polymorphic SNP markers, consisted of 14 linkage groups, and was approximately 728 cM in length (Lucas et al. 2011).

The Lucas et al. (2011) cowpea consensus genetic map vs. 4 is the most recent cowpea consensus genetic map, succeeding the vs. 2 (Muchero et al. 2009) and vs. 3 (Diop et al. 2012) maps. The vs. 4 cowpea consensus genetic map increased the marker density and improved the marker order using ten RIL populations and two F4 breeding populations (Lucas et al. 2011). The map is 680 cM in length and contains 1,107 markers with an average of 0.65 cM between markers (Lucas et al. 2011). The current SNP-based cowpea linkage map is included in the publicly available database HarvEST:Cowpea (http://harvest.ucr.edu) (www.harvest-web.org).

### Statistical analysis

MapQTL 5.0 software was used to conduct the QTL analyses (Van Ooijen 2004). QTLs were first analyzed using the Interval Mapping (IM) package to approximate putative QTLs; the closest marker to the putative QTL was used as a cofactor as a genetic background control for the MQM package of MapQTL5.0 (Van Ooijen 2004). The restricted MQM (rMQM) package was then used to determine the percentage of variance (*R* ^2^) explained by the QTL. A one-way analysis of variance using the Kruskal–Wallis (KW) package from MapQTL5.0 was used to confirm QTL loci (Van Ooijen 2004). Logarithm of the odds (LOD) thresholds were calculated using 1,000 permutations, resulting in a 95 % LOD threshold of approximately 2.1. 1-LOD and 2-LOD of the maximum peak were used to determine the left and right margins and the entire span of the QTL (Van Ooijen 2004). QTLs were visualized using MapChart 2.2 (Voorrips 2002).

### Synteny

Synteny was examined using EST-derived SNP markers from the cowpea consensus genetic map vs. 4 which were aligned to the soybean genome and functionally annotated using the most significant similarity using BLAST (Lucas et al. 2011). The cowpea consensus genetic map and syntenic relationships with model species can be viewed in the HarvEST:Cowpea database (http://harvest.ucr.edu) (www.harvest-web.org). Syntenic maps were drawn using HarvEST:Cowpea using a cut-off* e*-score value of −10. A minimum of five lines per linkage group was chosen to enable better viewing of syntenic relationships within the trait loci. Due to a limitation in the resolution, not all markers are presented in the screenshot images output from HarvEST:Cowpea. In order to view each individual marker, the linkage group must be magnified in the HarvEST:Cowpea database (http://harvest.ucr.edu) (www.harvest-web.org).

### Cowpea physical map

The cowpea physical map was developed using an advanced African breeding line IT93K-399-35 (http://phymap.ucdavis.edu/cowpea). Two bacterial artificial chromosome (BAC) clone libraries were developed using restriction enzymes *Hind*III and *Mbo*I (Amplicon Express, Pullman, WA, USA). Contigs were assembled using the snapshot method of DNA fingerprinting by Ming Cheng Luo at the University of California, Davis (Luo et al. 2003). The final physical map is an assembly of 43,717 BACs with an 11× genome depth of coverage (http://phymap.ucdavis.edu/cowpea).

## Results

The distribution of Fot race 4 phenotypes amongst the three cowpea populations was examined and is shown in Online Resources 2, 3, and 4. The mean disease values for the parental genotypes are labeled as such in the Figures.

### Fot race 4 QTL analysis in three cowpea populations

#### IT93K-503-1 × CB46

**Fig. 1:**
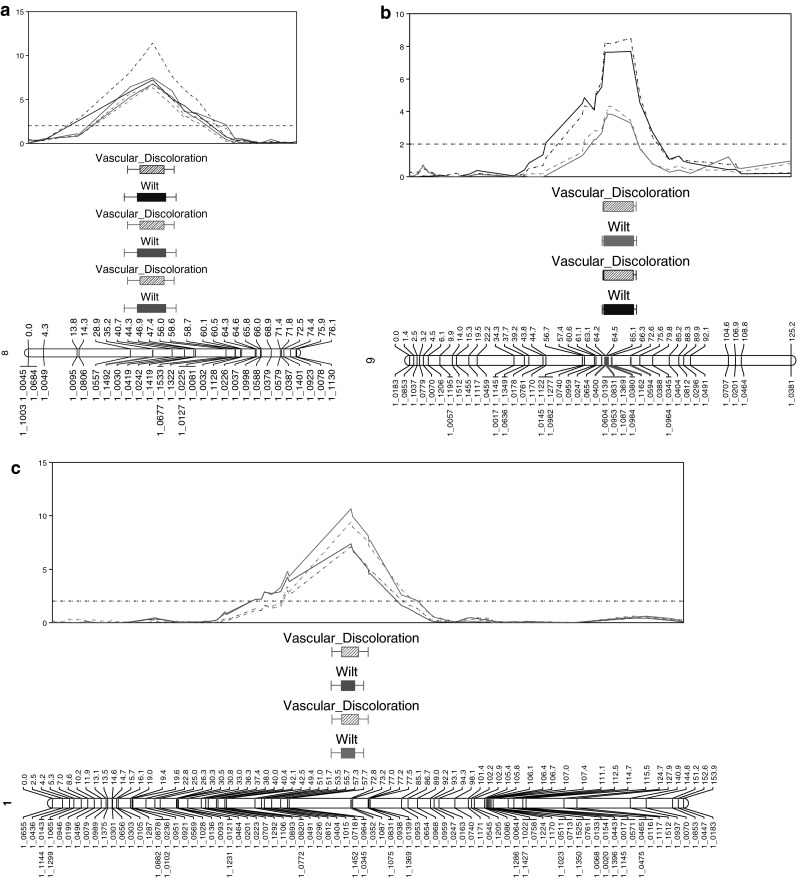
**a** Resistance to *Fusarium oxysporum* f.sp. *tracheiphilum* race 4: *Fot4*-*1* QTL in the IT93K-503-1 × CB46 population. The *Fot4*-*1* QTL mapped to linkage group 8. LOD scores for the first (2007), second (2010a), and third (2010b) experiments are plotted in *red*, *green*, and *blue*, respectively. *Solid colored lines* indicate the wilting/stunting phenotype and *broken colored lines* indicate the vascular discoloration phenotype. SNP marker 1_1492, which is *highlighted in red*, showed the most significant association with Fot race 4 over the three experiments. The LOD significance threshold of 2.0 is indicated by a *horizontal broken line*. **b** Resistance to *Fusarium oxysporum* f.sp. *tracheiphilum* race 4: *Fot4*-*2* QTL in the CB27 × 24-125B-1 population. The *Fot4*-*2* QTL mapped to linkage group 9. LOD scores are plotted in *blue* and *pink* for the first and second experiments, respectively. Over the two experiments, SNP markers 1_0594, 1_0984, 1_0380, and 1_1162 showed the most significant association with Fot race 4 resistance and are *highlighted in red* on the linkage group. The LOD significance threshold of 2.0 is indicated by a *horizontal broken line*. **c** Resistance to *Fusarium oxysporum* f.sp. *tracheiphilum* race 4: *Fot4*-*2* QTL in the CB27 × IT82E-18/Big Buff population. The *Fot4*-*2* QTL mapped to linkage group 1. LOD scores for the two experiments are plotted in *green* and *pink*. SNP marker 1_0352 was the most significant marker over both experiments and is *highlighted in red*. The LOD significance threshold of 2.0 is indicated by a *horizontal broken line*. (Color figure online)

IM and rMQM mapping using three phenotyping datasets identified one major QTL conferring resistance to Fot race 4 (Fig. 1a). The length of the locus, which is designated here as *Fot4*-*1*, spanned from 28.86 to 40.67 cM on linkage group 8 and was identified by SNP markers 1_0557, 1_1492, and 1_0030 (Fig. 1a, Online Resources 5 and 6). SNP marker 1_1492 was the most significant marker over all three experiments, accounting for 32.6 % (LOD 6.77), 32.7 % (LOD 7.48), and 32.7 % (LOD 7.22) of the phenotypic variance for the wilting/stunting phenotype and 30.3 % (LOD 6.74), 28.5 % (LOD 6.33), and 46.5 % (LOD 11.42) of the phenotypic variance for the vascular discoloration phenotype (Online Resource 6).

**Fig. 2:**
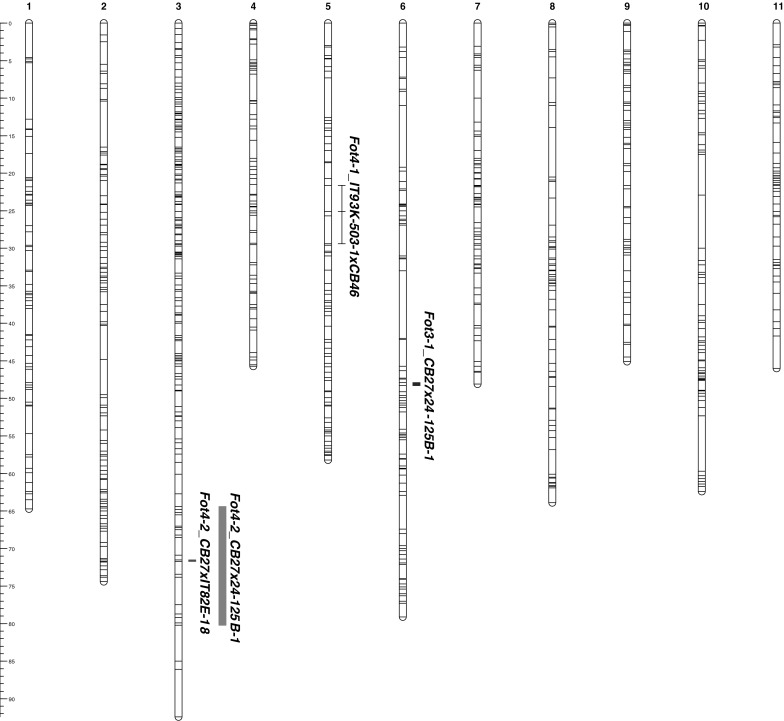
*Fusarium oxysporum* f.sp. *tracheiphilum* race 3 and race 4 resistance (*Fot3*-*1, Fot4*-*1*, and *Fot4*-*2)* on the cowpea consensus genetic map. QTLs which confer resistance to Fot race 3 and race 4 were positioned on the cowpea consensus genetic map vs. 4. *Fot3*-*1*, which confers resistance to Fot race 3 in the CB27 × 24-125B-1 population, was positioned on linkage group 6, spanning from 47.86 to 48.31 cM. *Fot4*-*1*, which confers resistance to Fot race 4 in the IT93K-503-1 × CB46 population, spanned from 21.57 to 29.40 cM on linkage group 5. The *Fot4*-*2* locus, which confers resistance to Fot race 4 in the CB27 × 24-125B-1 and CB27 × IT82E-18/Big Buff populations, was positioned on linkage group 3. Using the locus identified in the CB27 × IT82E-18/Big Buff population, the minimum distance of *Fot4*-*2* spanned from 71.52 to 71.75 cM. The maximum distance of *Fot4*-*2* identified in the CB27 × 24-125B-1 population spanned from 64.44 to 80.23 cM

The corresponding location of *Fot4*-*1* was positioned on the cowpea consensus genetic map using the significant markers identified in the QTL analysis. The *Fot4*-*1* locus spanned from 21.57 to 29.40 cM on the cowpea consensus genetic map linkage group 5 (Fig. 2, Online Resource 5). The length of the *Fot4*-*1* region on the cowpea consensus genetic map, 7.83 cM, was less than the estimated length of 11.81 cM identified on the IT93K-503-1 × CB46 individual map (Online Resource 5). However, the 7.83 cM estimated length of *Fot4*-*1* on the cowpea consensus map is most likely the more accurate estimate due to higher recombination utilizing the 12 constituent genetic maps (Lucas et al. 2011).

#### CB27 × 24-125B-1

Phenotyping datasets from two experiments were used to map Fot race 4 resistance which identified one locus, which we designated as *Fot4*-*2. Fot4*-*2* spanned 64.22–72.55 cM on linkage group 9 in the CB27 × 24-125B-1 population map (Fig. 1b, Online Resources 7 and 8). Marker 1_0594 was the most significant in the first experiment for both disease phenotypes, accounting for 37.6 % (LOD 7.69) variance for the wilt phenotype and 40.2 % (LOD 8.49) variance for the vascular discoloration phenotype (Online Resource 8). The second experiment identified SNP markers 1_0984, 1_0380, and 1_1162 as the most significant for both the wilting and the vascular discoloration phenotypes (Online Resource 8). SNP markers 1_0984, 1_0380, and 1_1162 were all in the same bin on the individual genetic map due to lack of recombination in the region (Online Resource 7); each marker thereby accounted for 32.3 % (LOD 3.82) of the phenotypic variance for wilting and 35.6 % (LOD 4.31) of the variance for the vascular discoloration phenotype (Online Resource 8).

Using the highly significant markers from the QTL study, *Fot4*-*2* was positioned on the cowpea consensus genetic map where it spanned the region from 64.44 to 80.23 cM on linkage group 3 (Fig. 2, Online Resource 7). The estimated length of 15.79 cM for the *Fot4*-*2* locus on the cowpea consensus genetic map is probably more accurate than the estimated 8.33 cM length on the individual map, particularly since eight out of eleven markers shared the same marker bin in the *Fot4*-*2* locus in the CB27 × 24-125B-1 population (Online Resource 7). Only 11 of the 26 markers in the *Fot4*-*2* locus on the cowpea consensus map were polymorphic in the CB27 × 24-125B-1 genetic map, which also may account for the smaller QTL length on the individual map (Online Resource 7).

#### CB27 × IT82E-18/Big Buff

Fot race 4 resistance was mapped using phenotyping datasets from two experiments. The QTL was identified on linkage group 1 of the individual map, spanning from 72.8 to 73.18 cM (total 0.38 cM) (Fig. 1c, Online Resource 7). SNP marker 1_0352 was the most significant over the two experiments, accounting for 27.1 % (LOD 10.66) and 19.6 % (LOD 7.34) of the phenotypic variance for wilting and 24 % (LOD 9.45) and 18.9 % (LOD 7.11) of the phenotypic variance for vascular discoloration (Online Resource 9).

The QTL observed in the CB27 × IT82E-18/Big Buff population was positioned on the cowpea consensus genetic map spanning from 71.52 to 71.75 cM (0.23 cM total distance) on linkage group 3 (Fig. 2, Online Resources 7 and 9). This locus overlapped with the position of *Fot4*-*2* identified in the CB27 × 24-125B-1 population (Fig. 2, Online Resource 7). The length of *Fot4*-*2,* 0.23 cM, on the cowpea consensus genetic map was similar to the length identified in the CB27 × IT82E-18/Big Buff individual map, 0.38 cM (Online Resource 7).

Subsequently, the *Fot4*-*2* locus was validated because it was identified in two different populations which share the same Fot race 4 resistance donor parent, CB27. Nevertheless, the *Fot4*-*2* locus identified in the two populations did not overlap perfectly on the cowpea consensus genetic map, because many of the markers that were significant in the CB27 × 24-125B-1 population (1_0594, 1_1162, 1_0380, and 1_0984) were not polymorphic in the CB27 × IT82E-18/Big Buff population, and vice versa. SNP marker 1_1087 was the only marker identified as being highly significant in both populations (Online Resources 7, 8, and 9). The maximum length of *Fot4*-*2* was defined by the QTL identified in the CB27 × 24-125B-1 population, which spanned from 64.44 to 80.23 cM on the cowpea consensus genetic map (Online Resource 7). However, as stated previously, there was much less recombination within the *Fot4*-*2* locus in the CB27 × 24-125B-1 population, indicated by several of the markers having the same cM position (Online Resource 7), which greatly limited the ability to narrow the QTL position. Considering that the *Fot4*-*2* locus identified in the CB27 × IT82E-18/Big Buff population was smaller due to rapid decrease in the significance threshold of the markers outside of the 2-LOD score (Online Resource 9), the shorter length spanning from 71.52 to 71.75 cM (0.23 cM distance) on the cowpea consensus genetic map may be a more accurate estimation of *Fot4*-*2*.

The results from this study established that *Fot4*-*1* and *Fot4*-*2* are independent of each other as observed on the cowpea consensus genetic map (Fig. 2). *Fot4*-*1* is positioned on linkage group 5, spanning 21.57 cM to 29.40 cM. The minimum distance of *Fot4*-*2* identified in CB27 × IT82E-18/Big Buff spanned from 71.52 to 71.75 cM on linkage group 3, while the maximum distance determined by the resistance locus identified in CB27 × 24-125B-1 spanned from 64.44 to 80.23 cM (Fig. 2). *Fot3*-*1,* which was previously identified in the CB27 (resistant) × 24-125B-1 (susceptible) population spanning from 49.92 to 50.49 cM on linkage group 1 of the individual genetic map and flanked by SNP markers 1_0860 and 1_1107 (Pottorff et al. 2012), was positioned on vs. 4 cowpea consensus genetic map where it spanned 47.86–48.31 cM region on linkage group 6 (Fig. 2). We therefore determined that both of the Fot race 4 resistance loci, *Fot4*-*1* and *Fot4*-*2,* are independent of the Fot race 3 locus, *Fot3*-*1* (Fig. 2).

The *Fot4*-*1* and *Fot4*-*2* loci were examined for markers which might co-segregate an allele with an associated disease-resistance phenotype using several cowpea genotypes with known reactions to Fot race 4. However, no such marker–trait associations were found for any of the markers in the *Fot4*-*1* or *Fot4*-*2* loci. This suggests that the density of markers in the Fot race 4 resistance regions was not high enough to find a marker closely linked with resistance and neither *Fot4*-*1* nor *Fot4*-*2* could be narrowed further.

### Synteny of Fot race 4 loci with *G. max*

**Fig. 3:**
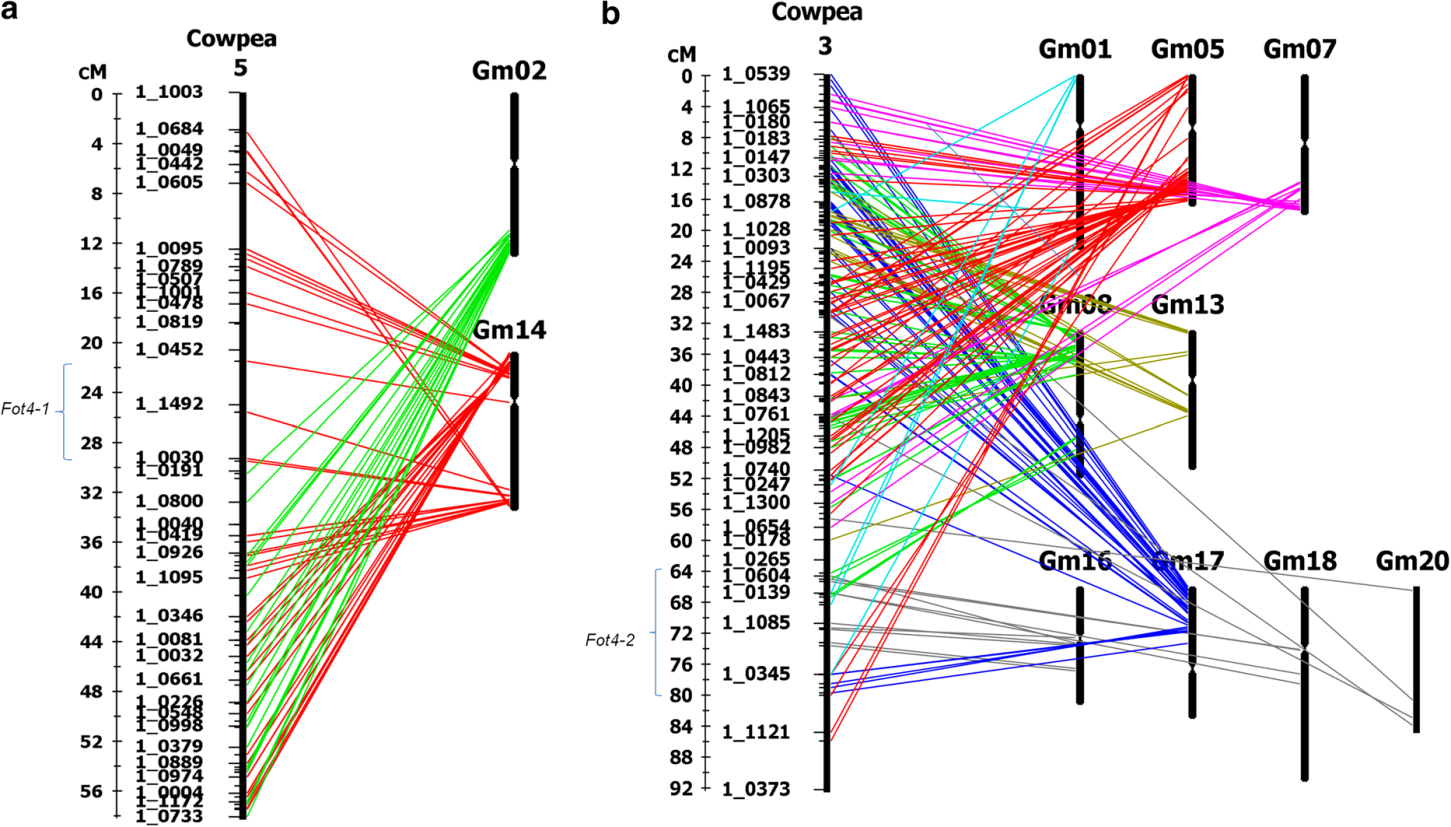
**a** Synteny of *Fot4*-*1* with *G. max* chromosome 14. Synteny was examined for the *Fot4*-*1* locus between cowpea and *G. max* using EST-derived SNP markers previously BLASTed and aligned to the sequenced genomes. *Fot4*-*1* spans from 21.57 to 29.40 cM on the cowpea consensus genetic map linkage group 5 and was syntenic at a macro and micro scale with soybean chromosome 14. The *Fot4*-*1* syntenic locus in soybean was identified by soybean orthologs to cowpea SNP markers 1_0557, 1_0662, 1_0986, and 1_0030 and spanned from soybean locus Glyma14g15370 to Glyma14g36620. Three soybean disease-resistance genes, Glyma14g17910, Glyma14g23930, and Glyma14g34880, were observed in the syntenic locus and were considered as orthologous disease-resistance candidate genes for the *Fot4*-*1* locus. Glyma14g17910 and Glyma14g23930 were both annotated as TIR-NBS–LRR genes and Glyma14g34880 was annotated as a leucine-rich repeat protein kinase. **b** Synteny of *Fot4*-*2* locus with *G. max* chromosomes 16 and 18. Synteny was examined for the *Fot4*-*2* locus between cowpea and *G. max* using EST-derived SNP markers previously BLASTed and aligned to the sequenced genomes. The *Fot4*-*2* locus, which spanned 64.44–80.23 cM on cowpea consensus genetic map linkage group 3, was determined to be co-linear with soybean chromosomes 16 and 18. The syntenic region in soybean chromosome 16 spanned from soybean locus Glyma16g15790 to Glyma16g23710, where two soybean disease-resistance genes, Glyma16g17380 and Glyma16g22620, were observed. Glyma16g17380 was annotated as a leucine-rich repeat protein kinase and Glyma16g22620 was annotated as a TIR–NBS–LRR disease-resistance gene. The syntenic *Fot4*-*2* region of soybean chromosome 18 spanned from soybean locus Glyma18g18980 to Glyma18g38670, which corresponded to 65.16–66.99 cM of the *Fot4*-*2* locus. However, the syntenic region preceded the most significant region of the *Fot4*-*2* locus, and no candidate genes were observed

The *Fot4*-*1* and *Fot4*-*2* loci in cowpea were compared with the soybean genome to determine if a syntenic relationship exists. A high co-linearity of the *Fot4*-*1* or *Fot4*-*2* loci with the sequenced soybean genome may enable the identity of candidate disease-resistance genes to be determined. The *Fot4*-*1* locus in cowpea was compared with the soybean genome, which was found to be highly co-linear with soybean chromosome 14 (Fig. 3a; Table 1). Soybean genes orthologous to cowpea SNP markers identified the syntenic locus spanning from soybean locus Glyma14g15370 to Glyma14g36620, which corresponded to the 21.57–29.40 cM region in the *Fot4*-*1* locus (Table 1). The orthologous soybean genes were in the same order as the SNP markers in the cowpea consensus genetic map with the exception of the ortholog for SNP 1_ 1492, which was missing (Online Resource 5, Table 1). The cowpea SNP markers near to the *Fot 4*-*1* locus, 1_0557 and 1_0662, were examined on the soybean genome browser on the Phytozome webpage for known disease-resistance genes (http://www.phytozome.net). Although cowpea markers were not precisely positioned within soybean genes, three disease-resistance soybean genes were observed in the syntenic *Fot4*-*1* locus, viz. Glyma14g17910, Glyma14g23930, and Glyma14g34880, and were considered as orthologous disease-resistance candidate genes (Table 1). Soybean loci Glyma14g17910 and Glyma14g23930 were both annotated as Toll/interleukin1-like receptor nucleotide-binding site leucine-rich repeat (TIR–NBS–LRR) genes (Table 1). Glyma14g34880 was annotated as a leucine-rich repeat protein kinase (Table 1).

**Table 1:** Synteny of *Fot4*-*1* with *G. max* chromosome 14

*G. max* locus	*G. max* location	Phytozome annotation	Cowpea locus	LG	cM
Glyma14g15370	Gm14: 16294823–16294996	Ribosomal protein	1_0557	5	21.57
Glyma14g17910	Gm14: 19987489–19988368	TIR–NBS–LRR disease resistance protein	N/A	N/A	N/A
Glyma14g23930	Gm14: 28439271–28446522	TIR–NBS–LRR disease resistance protein	N/A	N/A	N/A
Glyma14g34880	Gm14: 43590997–43594201	Leucine-rich repeat serine/threonine protein kinase	N/A	N/A	N/A
Glyma14g35330	Gm14: 44224418–44225596	Phosphate-responsive protein	1_0662	5	25.70
Glyma14g35340	Gm14: 44234374–44235568	Phosphate-responsive protein	1_0986	5	25.70
Glyma14g36620	Gm14: 45983440–45985244	60S ribosomal protein	1_0030	5	29.40

*LG* linkage group

The *Fot4*-*2* locus was examined for a possible syntenic relationship with the soybean genome, in which a co-linear relationship at the macro and micro level was observed with soybean chromosomes 16 and 18 (Fig. 3b; Table 2). The syntenic region in soybean chromosome 16 spanned from soybean locus Glyma16g15790 to Glyma16g23710, corresponding to the 64.78–73.79 cM region of the *Fot4*-*2* locus on the cowpea consensus genetic map (Online Resource 7, Table 2). The soybean genes that were orthologous to cowpea EST-derived SNP markers were in the same marker order as in the cowpea consensus genetic map, with the exception of the soybean ortholog of SNP 1_0604 (64.78 cM), which preceded the corresponding 71.52–73.79 cM region (Online Resource 7). The syntenic region spanning between orthologous soybean genes to cowpea SNP markers 1_1087 and 1_0984 was examined on the soybean genome browser on the Phytozome webpage for known disease-resistance genes (http://www.phytozome.net). Two disease-resistance soybean loci were observed in the syntenic region: Glyma16g17380, which was annotated as a leucine-rich repeat protein kinase, and Glyma16g22620, which was annotated as a TIR–NBS–LRR disease-resistance gene (Table 2). Additionally, a cluster of five leucine-rich repeat protein kinases was observed flanked between soybean genes orthologous to SNP markers 1_0380 and 1_1162, which corresponded to 73.42–73.79 cM of the *Fot4*-*2* locus (Table 2). Due to the close proximity to the most significant region of *Fot4*-*2* (71.52–71.75 cM), all seven of the soybean genes were considered as orthologous candidate genes for the *Fot4*-*2* locus.

**Table 2:** Synteny of *Fot4*-*2* with *G. max* chromosomes 16 and 18

*G. max* chromosome	*G. max* locus	*G. max* location	Phytozome annotation	Cowpea locus	LG	cM
16	Glyma16g15790	Gm16: 16709092–16712991	Unknown function	1_1087	3	71.52
16	Glyma16g17190	Gm16: 18531838–18537592	Pectinacetylesterase	1_0604	3	64.78
16	Glyma16g17380	Gm16: 18846672–18849661	Leucine-rich repeat serine/threonine protein kinase	N/A	N/A	N/A
16	Glyma16g17680	Gm16: 19294324–19298758	NmrA-like family	1_0352	3	71.75
16	Glyma16g22620	Gm16: 26094883–26102980	TIR–NBS–LRR disease resistance protein	N/A	N/A	N/A
16	Glyma16g23120	Gm16: 26788171–26791817	Aspartyl protease	1_0984	3	73.42
16	Glyma16g23230	Gm16: 26958715–26960456	Skp1 family protein	1_0380	3	73.42
16	Glyma16g23430	Gm16: 27190882–27193074	Leucine-rich repeat serine/threonine protein kinase	N/A	N/A	N/A
16	Glyma16g23450	Gm16: 27214661–27216604	Leucine-rich repeat serine/threonine protein kinase	N/A	N/A	N/A
16	Glyma16g23500	Gm16: 27258637–27261832	Leucine-rich repeat serine/threonine protein kinase	N/A	N/A	N/A
16	Glyma16g23530	Gm16: 27327094–27329549	Leucine-rich repeat serine/threonine protein kinase	N/A	N/A	N/A
16	Glyma16g23560	Gm16: 27364956–27367998	Leucine-rich repeat serine/threonine protein kinase	N/A	N/A	N/A
16	Glyma16g23710	Gm16: 27560220–27563581	Oxidoreductase	1_1162	3	73.79
18	Glyma18g18980	Gm18: 20554229–20556614	BURP domain	1_0400	3	65.51
18	Glyma18g19050	Gm18: 20735387–20738374	Alcohol dehydrogenase	1_0444	3	65.16
18	Glyma18g24740	Gm18: 28509583–28511103	No functional annotation	1_1369	3	66.99
18	Glyma18g27710	Gm18: 31737329–31742252	Serine hydroxymethyltransferase	1_0139	3	66.99
18	Glyma18g38670	Gm18: 46319160–46324669	Alcohol dehydrogenase	1_0207	3	66.99

*LG* linkage group

The syntenic *Fot4*-*2* region of soybean chromosome 18 spanned from soybean locus Glyma18g18980 to Glyma18g38670, where five out of six soybean orthologs for cowpea SNP markers corresponded to 65.16–66.99 cM of the *Fot4*-*2* region on cowpea linkage group 3 (Table 2, Online Resource 7). The soybean genes orthologous to cowpea SNP markers were in the same linear order as on the cowpea genetic map; however, this syntenic locus preceded the most significant region of the *Fot4*-*2* locus, 64.78–73.79 cM, and no disease-resistance candidate genes were observed or expected (Table 2).

### *Fot4*-*1* and *Fot4*-*2* loci on the cowpea physical map

The cowpea physical map (http://phymap.ucdavis.edu/cowpea), which has been anchored to the cowpea consensus genetic map via SNP markers, was used to identify contigs which span the physical regions of *Fot4*-*1* and *Fot4*-*2*.

Significant markers from the *Fot4*-*1* locus and closely linked markers from the cowpea consensus genetic map vs. 4 identified two cowpea BAC contigs, contig77 and contig417, which incompletely span the *Fot4*-*1* region (Online Resource 5). The only significant SNP marker, 1_0030, identified in the *Fot4*-*1* locus was identified in contig417 within BAC clones CH027H24 and CH035P21 on the cowpea physical map (Online Resource 5). SNP 1_0662, which is linked with marker 1_0030 on the cowpea consensus genetic map, was identified in BAC contig 77 within BAC clone CH095K15 (Online Resource 5). The other SNP markers within the *Fot4*-*1* locus, 1_0557 and 1_1492, were not observed in the cowpea physical map and are probably not present in the African breeding line IT97K-499-35 which was used to create the cowpea physical map.

SNP markers from the *Fot4*-*2* locus on the cowpea consensus genetic map identified seven contigs and nine BAC clones which partially span the locus on the cowpea physical map. The significant markers for the *Fot4*-*2* region resulting from the QTL analysis identified four contigs and five BAC clones in CB27 × 24-125B-1 and three contigs and four BACs in CB27 × IT82E-18/Big Buff (Online Resource 7). The most significant marker identified in the CB27 × IT82E-18/Big Buff population, 1_0352, was identified in contig1094, BAC clone CM052M22 (Online Resource 7). Since the *Fot4*-*1* and *Fot4*-*2* loci could not be narrowed further and the physical map spanning both regions was incomplete, the physical to genetic map distance was not analyzed.

## Discussion

This study has identified two independent loci, *Fot4*-*1* and *Fot4*-*2*, which confer resistance against *F. oxysporum* f.sp. *tracheiphilum* race 4 in cowpea. These two resistance loci were inherited from two different cowpea genotypes which differ in origin; *Fot4*-*1* is derived from an African breeding line, IT93K-503-1, and *Fot4*-*2* is derived from a US blackeye dry grain cultivar, CB27. In addition, *Fot4*-*1*, *Fot4*-*2*, and the previously identified *Fot3*-*1* were positioned on the cowpea consensus genetic map, confirming that these loci which confer race-specific resistance are independent of each other. The *Fot4*-*2* QTL was validated since it was identified in two independent populations, whose resistance locus was derived from the same CB27 resistant parent. The physical locations of *Fot4*-*1* and *Fot4*-*2* were roughly identified on the cowpea physical map, which will enable the generation of tightly linked markers which segregate with Fot race 4 resistance. Identification of the two independent Fot race 4 loci will enable gene pyramiding which may promote the durability of Fot race 4 resistance in future cowpea cultivars.

The candidate gene discovery utilizing synteny between cowpea and soybean identified TIR–NBS–LRR proteins and leucine-rich repeat serine/threonine protein kinases in the soybean syntenic regions of the *Fot4*-*1* and *Fot4*-*2* loci. Previous reports of resistance to Fusarium have indicated that the resistance is a monogenic trait with dominant expression (Zink and Thomas 1990; Rubio et al. 2003; McGrath et al. 1987; Scott and Jones 1989; Sarfatti et al. 1991). This profile conforms to the classic gene-for-gene model of Flor (1971) in which the pathogen and host express complementary dominant genes, avirulence and resistance genes, with the alteration or loss of either resulting in a compatible interaction and disease. Most disease-resistance genes fitting this profile have an NBS–LRR motif which, depending on the N-terminus design, has homology with the TIR domain (TIR–NBS–LRR) (Meyers et al. 1999; Pan et al. 2000) or a coiled-coil motif (CC-NBS–LRR or non TIR–NBS–LRR) (Pan et al. 2000). Of the two cloned genes which confer resistance to Fusarium wilt, both the *I*-*2* locus for resistance to *F. oxysporum* f.sp. *lycopersici* (Fol) race 2 in tomato (Simons et al. 1998) and the *Fom*-*2* locus for resistance to *F. oxysporum* f.sp *melonis* (Fom) in melon (Joobeur et al. 2004) were found to be CC-NBS–LRR genes.

Beyond the conserved NBS–LRR structure, other disease-resistance genes belonging to the receptor-like kinase (RLK) family, whose proteins span the plasma membrane and respond to extracellular signals (Geer et al. 1994). The majority of RLKs have serine/threonine kinases and LRR motifs (Becraft 1998) and include genes *PBS1*, *Pti, Pto*, and *Xa21* which confer resistance to bacterial pathogens in *Arabidopsis*, tomato, and rice (Shiu and Bleecker 2001; Song et al. 1995) and *Lrk10* which confers resistance to the fungus *Puccinia recondite* in wheat (Feuillet et al. 1997). Furthermore, the *I*-*3* locus which confers resistance to *F. oxysporum* f.sp. *lycopersici* race 3 in tomato was determined to be positioned within a large cluster of S-locus receptor-like kinases (Hemming et al. 2004), and recently we sequenced a BAC clone in the *Fot3*-*1* locus, which had four cowpea sequences with homology to leucine-rich repeat serine/threonine kinase receptors (LRR–STKR) (Pottorff et al. 2012). Based on these reports and our current findings, it is a good possibility that LRR–STKRs are the resistance genes responsible in the cowpea–Fusarium pathovar system.

Currently, the sequencing of BAC clones within the minimum tiling path of each BAC contig of the cowpea physical map is underway. This, combined with identification of markers more closely linked with the *Fot4*-*1* and *Fot4*-*2* loci, will enable the direct identification of cowpea disease-resistance candidate genes. A more immediate practical outcome of this study is the development of molecular markers closely linked to the *Fot4*-*1* and *Fot4*-*2* loci. These markers can be used for indirect selection of resistance in breeding schemes such as pedigree backcrossing and marker-assisted recurrent selection.

## Electronic supplementary material

Below is the link to the electronic supplementary material.

Online Resource 1 *Fusarium oxysporum* f.sp. *tracheiphilum* race 4 phenotyping for vascular discoloration symptoms. The severity of the vascular discoloration disease symptom was evaluated on a zero to five rating score. A rating of zero indicated a healthy plant with no signs of disease, 1 indicated approximately 10% of the plant with disease symptoms, 2 indicated 25% , 3 indicated 50%, 4 indicated 75% and 5 indicated 100% of the plant with disease symptoms (PPTX 403 kb)

Online Resource 2 Frequency distribution of the Fot race 4 phenotypes on over 100 RIL s in the IT93K-503-1xCB46 population. The mean resistance values for the parents are indicated by the arrows. Figures 1a and 1b belong to experiment 1; Figures 2a and 2b to experiment 2 and Figures 3a and 3b to the third experiment (PPTX 591 kb)

Online Resource 3 Frequency distribution of the Fot race 4 phenotypes on over 100 RIL s in the CB27x24-125B-1 population. The mean resistance values for the parents are indicated by the arrows. Figures 1a and 1b belong to the first experiment and Figures 2a and 2b belong to the second experiment (PPTX 421 kb)

Online Resource 4 Frequency distribution of the Fot race 4 phenotypes on over 100 RIL s in the CB27xIT82E-18 population. The mean resistance values for the parents are indicated by the arrows. Figures 1a and 1b belong to the first experiment and Figures 2a and 2b belong to the second experiment (PPTX 451 kb)

Online Resource 5 *Fot4-1* in the IT93K-503-1 x CB46 population, the cowpea consensus genetic map and the cowpea physical map (PPTX 63 kb)

Online Resource 6 QTL analysis of *Fot4-1* in the IT93K-503-1 x CB46 population (PPTX 56 kb)

Online Resource 7 *Fot4-2* locus in the CB27 x 24-125B-1 and CB27 x IT82E-18/Big Buff populations, the cowpea consensus genetic map and the cowpea physical map (PPTX 63 kb)

Online Resource 8 QTL analysis of *Fot4-2* in the CB27 x 24-125B-1 population (PPTX 58 kb)

Online Resource 9 QTL analysis of *Fot4-2* in the CB27 x IT82E-18 /Big Buff population (PPTX 54 kb)

## Abbreviations

BAC: Bacterial artificial chromosome
bp: Base pairs
CC-NBS–LRR: Coiled-coil nucleotide binding site–leucine-rich repeat
cM: CentiMorgan
EST: Expressed sequence tag
LG: Linkage group
LOD: Logarithm (base 10) of odds
LRR: Leucine-rich repeat
Mb: Megabases
NB: Nucleotide binding
NBS–LRR: Nucleotide-binding site–leucine-rich repeat
PDA: Potato dextrose agar
QTL: Quantitative trait locus
RGA: Resistance gene analog
RIL: Recombinant inbred line
SNP: Single nucleotide polymorphism
TIR: Toll/interleukin-1 receptor
