# Supplementary material for: Genetic mapping, synteny, and physical location of two loci for Fusarium oxysporum f. sp. tracheiphilum race 4 resistance in cowpea [Vignaunguiculata (L.) Walp]
Source: Mol Breed. 2013 Dec 13;33(4):779–91. doi: 10.1007/s11032-013-9991-0 (PMC3956937; doi:10.1007/s11032-013-9991-0)
Supplement: Supplementary file 1 — Online Resource 1 Fusarium oxysporum f.sp. tracheiphilum race 4 phenotyping for vascular discoloration symptoms. The severity of the vascular discoloration disease symptom was evaluated on a zero to five rating score. A rating of zero indicated a healthy plant with no signs of disease, 1 indicated approximately 10% of the plant with disease symptoms, 2 indicated 25% , 3 indicated 50%, 4 indicated 75% and 5 indicated 100% of the plant with disease symptoms (PPTX 403 kb) [file 11032_2013_9991_MOESM1_ESM.pptx]

## Slide 1
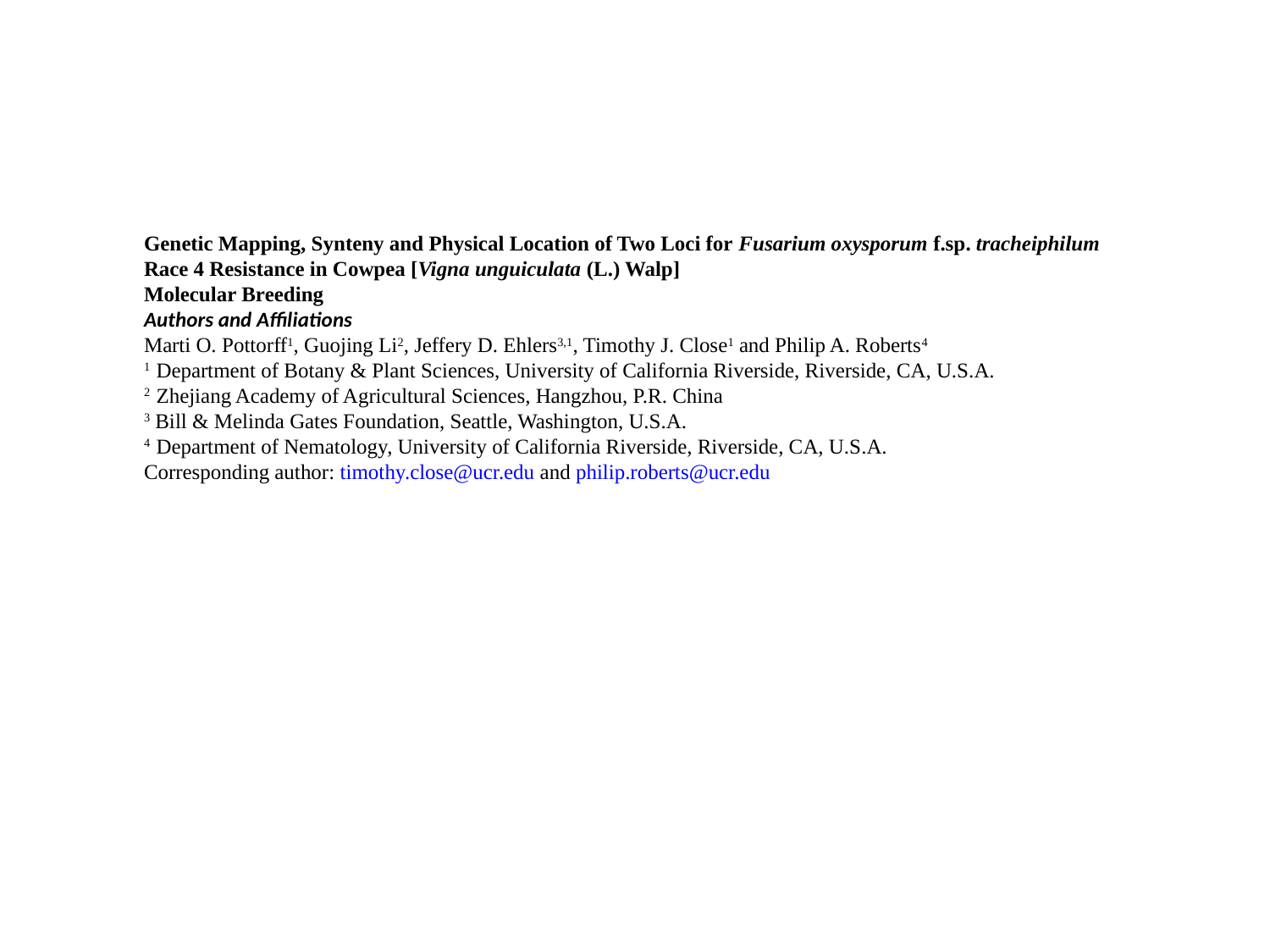

Genetic Mapping, Synteny and Physical Location of Two Loci for Fusarium oxysporum f.sp. tracheiphilum
Race 4 Resistance in Cowpea [Vigna unguiculata (L.) Walp]
Molecular Breeding
Authors and Affiliations
Marti O. Pottorff1, Guojing Li2, Jeffery D. Ehlers3,1, Timothy J. Close1 and Philip A. Roberts4
1 Department of Botany & Plant Sciences, University of California Riverside, Riverside, CA, U.S.A.
2 Zhejiang Academy of Agricultural Sciences, Hangzhou, P.R. China
3 Bill & Melinda Gates Foundation, Seattle, Washington, U.S.A.
4 Department of Nematology, University of California Riverside, Riverside, CA, U.S.A.
Corresponding author: timothy.close@ucr.edu and philip.roberts@ucr.edu

## Slide 2
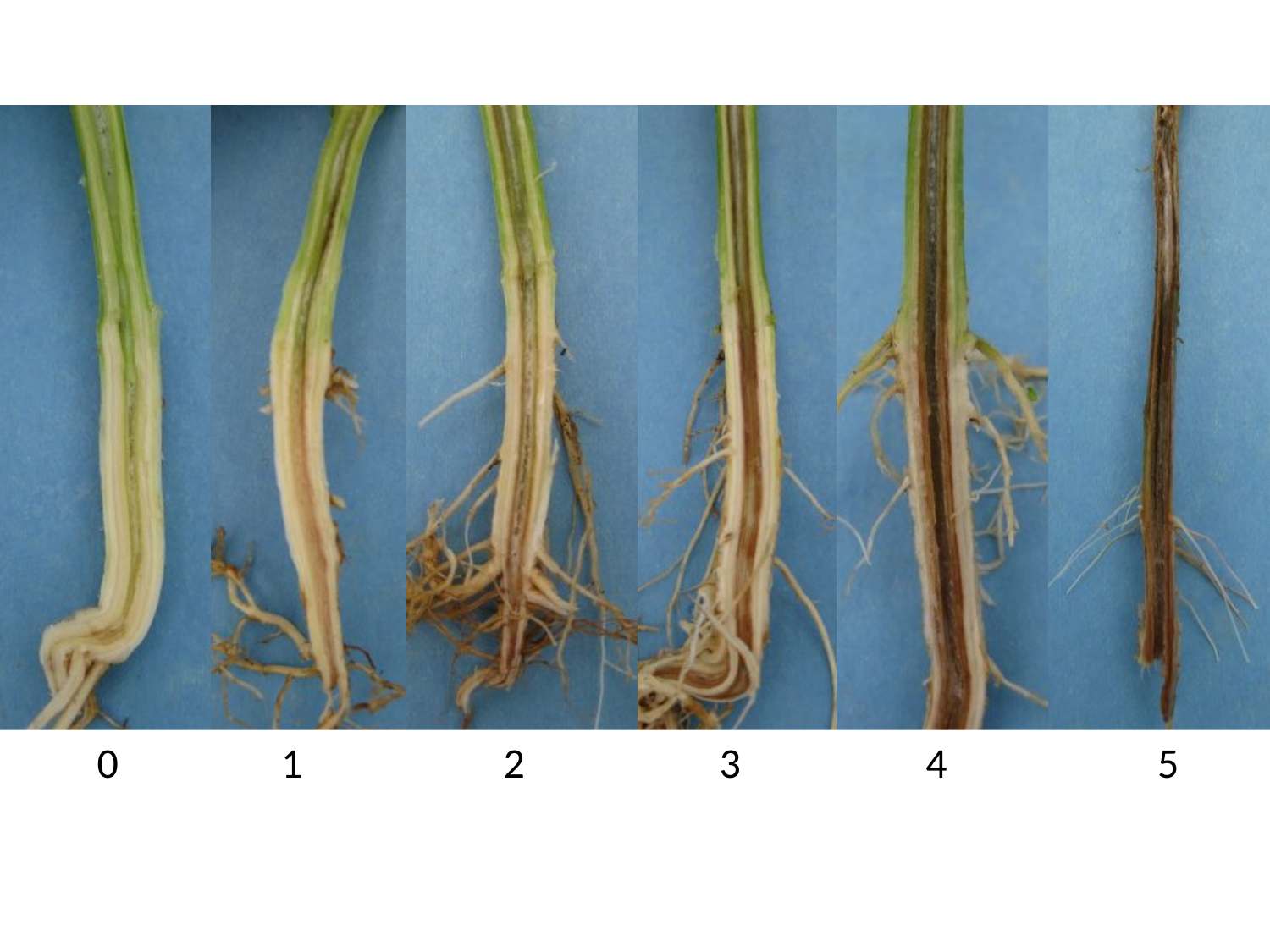

0	 1	 2	 3	 4	 5
