# Supplementary material for: Genetic mapping, synteny, and physical location of two loci for Fusarium oxysporum f. sp. tracheiphilum race 4 resistance in cowpea [Vignaunguiculata (L.) Walp]
Source: Mol Breed. 2013 Dec 13;33(4):779–91. doi: 10.1007/s11032-013-9991-0 (PMC3956937; doi:10.1007/s11032-013-9991-0)

## Slide 1
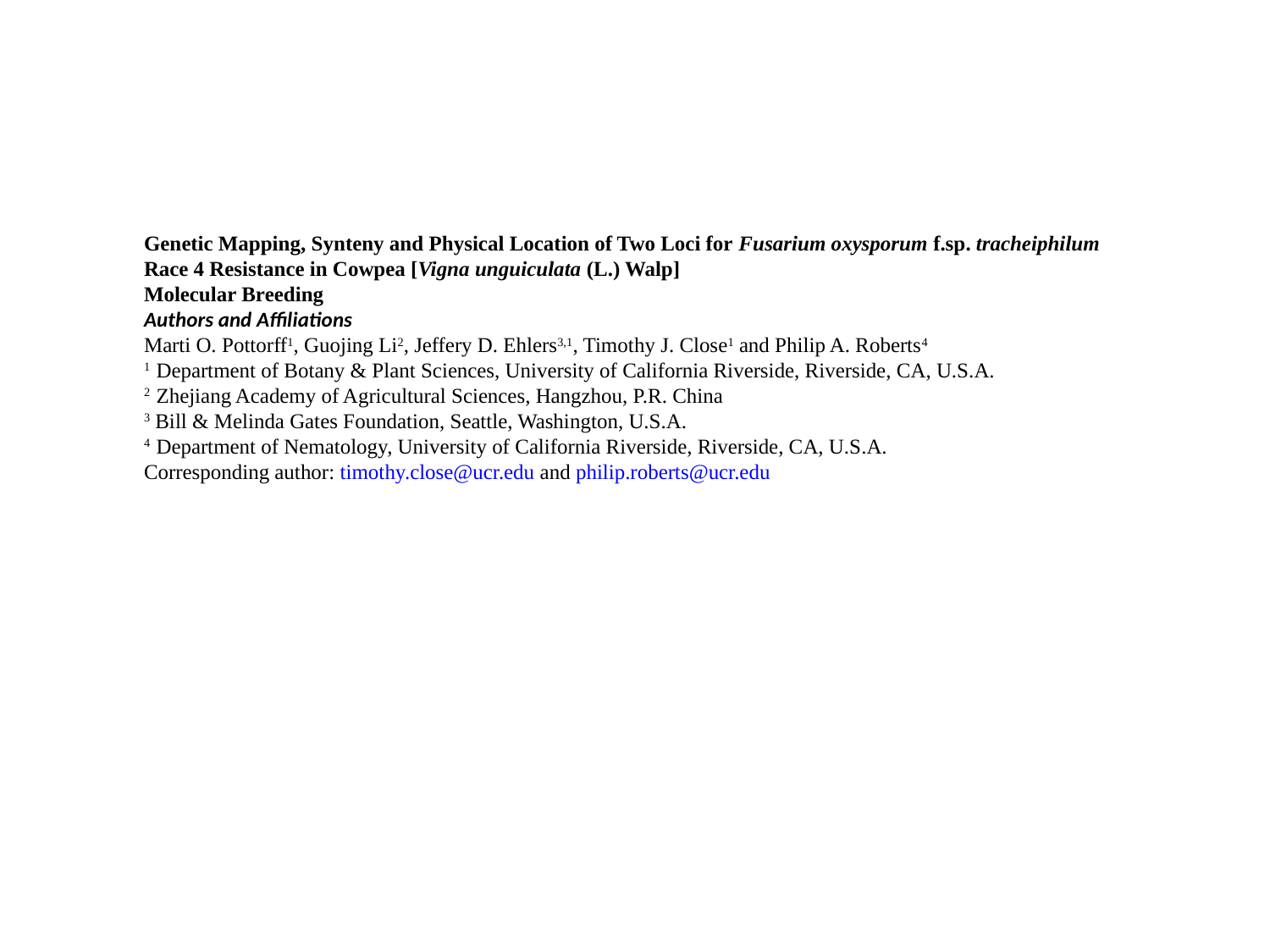

Genetic Mapping, Synteny and Physical Location of Two Loci for Fusarium oxysporum f.sp. tracheiphilum
Race 4 Resistance in Cowpea [Vigna unguiculata (L.) Walp]
Molecular Breeding
Authors and Affiliations
Marti O. Pottorff1, Guojing Li2, Jeffery D. Ehlers3,1, Timothy J. Close1 and Philip A. Roberts4
1 Department of Botany & Plant Sciences, University of California Riverside, Riverside, CA, U.S.A.
2 Zhejiang Academy of Agricultural Sciences, Hangzhou, P.R. China
3 Bill & Melinda Gates Foundation, Seattle, Washington, U.S.A.
4 Department of Nematology, University of California Riverside, Riverside, CA, U.S.A.
Corresponding author: timothy.close@ucr.edu and philip.roberts@ucr.edu

## Slide 2
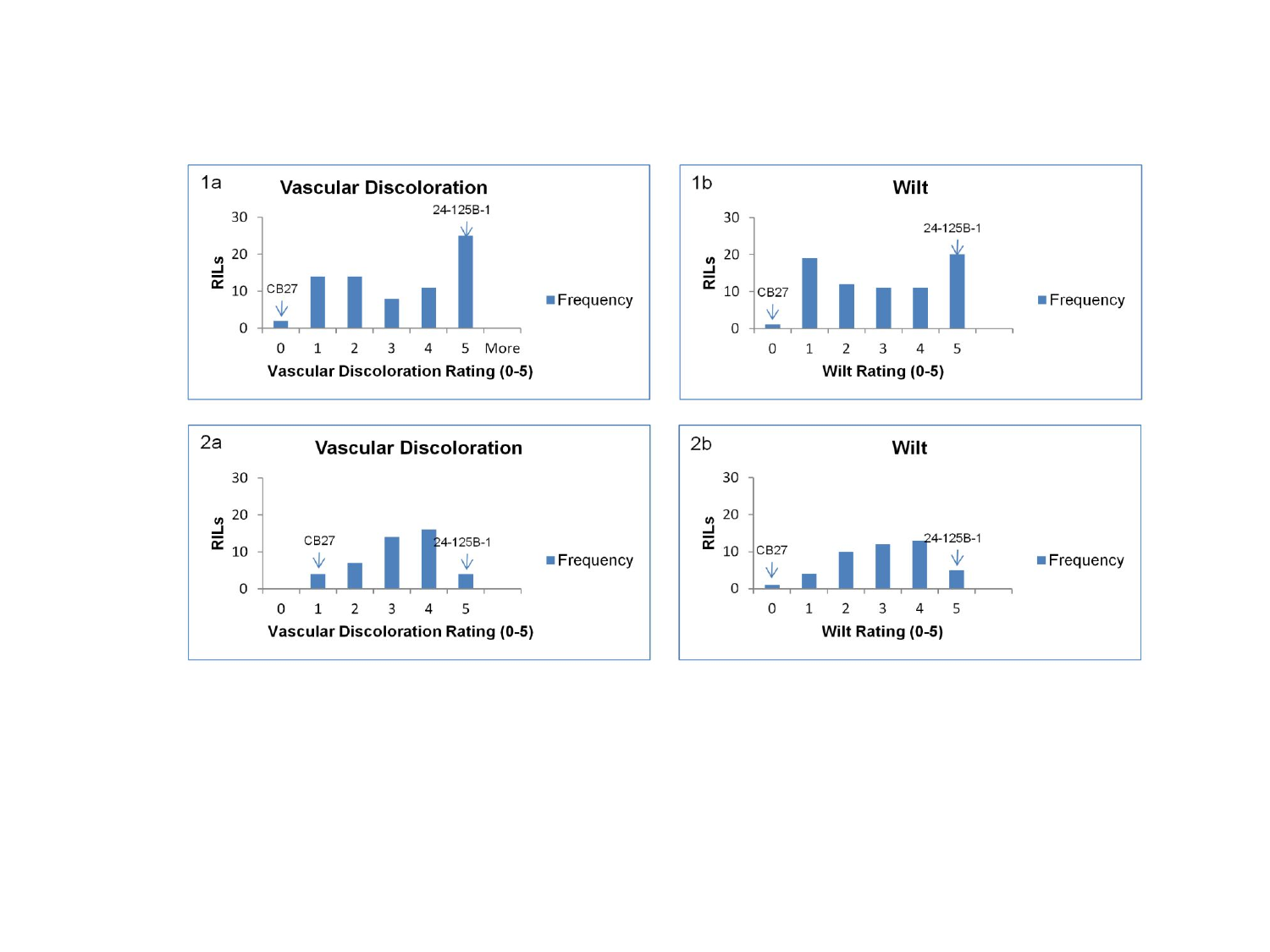

Supplement: Supplementary file 3 — Online Resource 3 Frequency distribution of the Fot race 4 phenotypes on over 100 RIL s in the CB27x24-125B-1 population. The mean resistance values for the parents are indicated by the arrows. Figures 1a and 1b belong to the first experiment and Figures 2a and 2b belong to the second experiment (PPTX 421 kb) [file 11032_2013_9991_MOESM3_ESM.pptx]
