# Supplementary material for: Genetic mapping, synteny, and physical location of two loci for Fusarium oxysporum f. sp. tracheiphilum race 4 resistance in cowpea [Vignaunguiculata (L.) Walp]
Source: Mol Breed. 2013 Dec 13;33(4):779–91. doi: 10.1007/s11032-013-9991-0 (PMC3956937; doi:10.1007/s11032-013-9991-0)
Supplement: Supplementary file 5 — Online Resource 5 Fot4-1 in the IT93K-503-1 x CB46 population, the cowpea consensus genetic map and the cowpea physical map (PPTX 63 kb) [file 11032_2013_9991_MOESM5_ESM.pptx]

## Slide 1
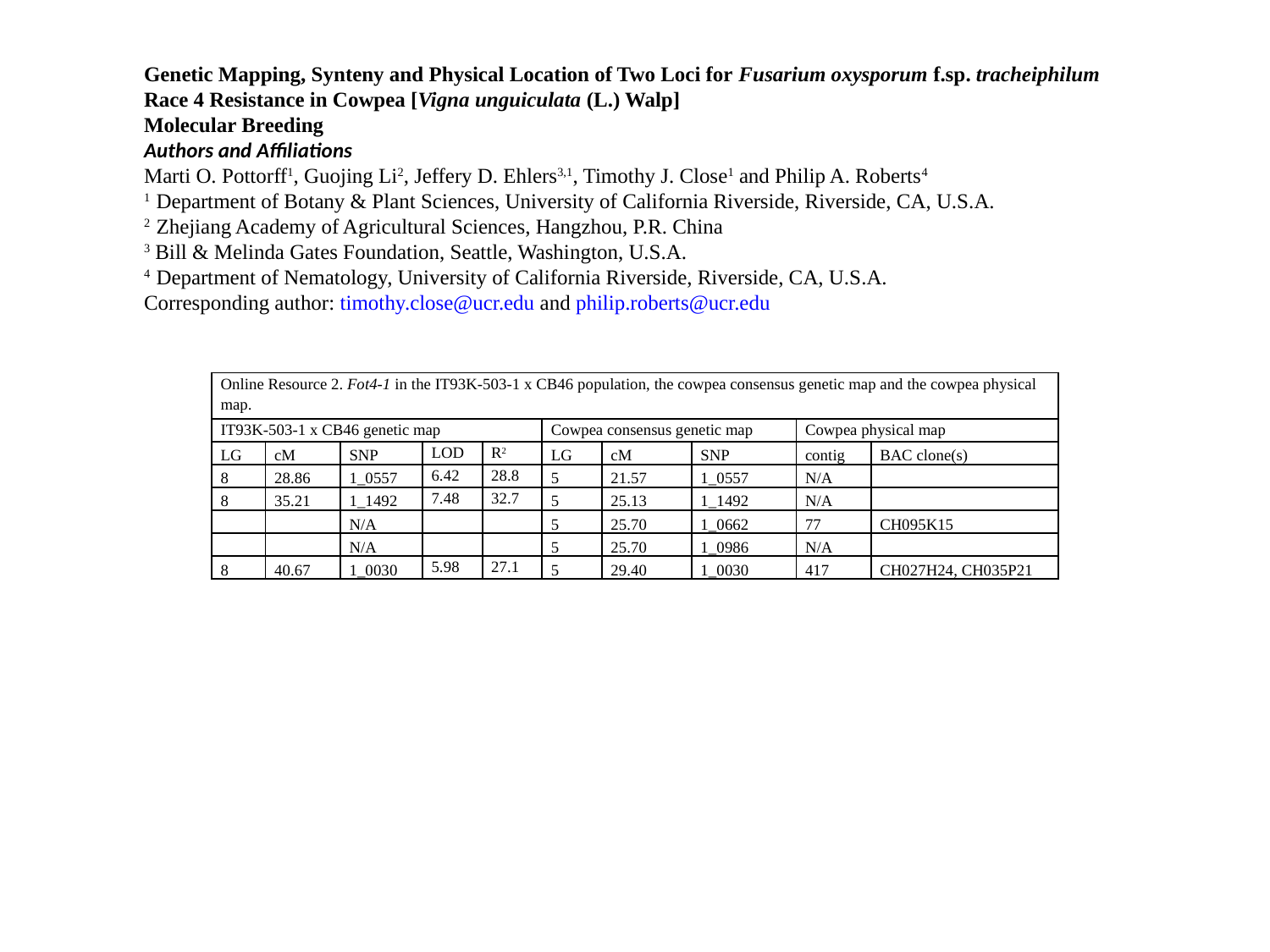

Genetic Mapping, Synteny and Physical Location of Two Loci for Fusarium oxysporum f.sp. tracheiphilum
Race 4 Resistance in Cowpea [Vigna unguiculata (L.) Walp]
Molecular Breeding
Authors and Affiliations
Marti O. Pottorff1, Guojing Li2, Jeffery D. Ehlers3,1, Timothy J. Close1 and Philip A. Roberts4
1 Department of Botany & Plant Sciences, University of California Riverside, Riverside, CA, U.S.A.
2 Zhejiang Academy of Agricultural Sciences, Hangzhou, P.R. China
3 Bill & Melinda Gates Foundation, Seattle, Washington, U.S.A.
4 Department of Nematology, University of California Riverside, Riverside, CA, U.S.A.
Corresponding author: timothy.close@ucr.edu and philip.roberts@ucr.edu
| Online Resource 2. Fot4-1 in the IT93K-503-1 x CB46 population, the cowpea consensus genetic map and the cowpea physical map. | | | | | | | | | |
| --- | --- | --- | --- | --- | --- | --- | --- | --- | --- |
| IT93K-503-1 x CB46 genetic map | | | | | Cowpea consensus genetic map | | | Cowpea physical map | |
| LG | cM | SNP | LOD | R2 | LG | cM | SNP | contig | BAC clone(s) |
| 8 | 28.86 | 1\_0557 | 6.42 | 28.8 | 5 | 21.57 | 1\_0557 | N/A | |
| 8 | 35.21 | 1\_1492 | 7.48 | 32.7 | 5 | 25.13 | 1\_1492 | N/A | |
| | | N/A | | | 5 | 25.70 | 1\_0662 | 77 | CH095K15 |
| | | N/A | | | 5 | 25.70 | 1\_0986 | N/A | |
| 8 | 40.67 | 1\_0030 | 5.98 | 27.1 | 5 | 29.40 | 1\_0030 | 417 | CH027H24, CH035P21 |
