# Supplementary material for: Genetic mapping, synteny, and physical location of two loci for Fusarium oxysporum f. sp. tracheiphilum race 4 resistance in cowpea [Vignaunguiculata (L.) Walp]
Source: Mol Breed. 2013 Dec 13;33(4):779–91. doi: 10.1007/s11032-013-9991-0 (PMC3956937; doi:10.1007/s11032-013-9991-0)
Supplement: Supplementary file 6 — Online Resource 6 QTL analysis of Fot4-1 in the IT93K-503-1 x CB46 population (PPTX 56 kb) [file 11032_2013_9991_MOESM6_ESM.pptx]

## Slide 1
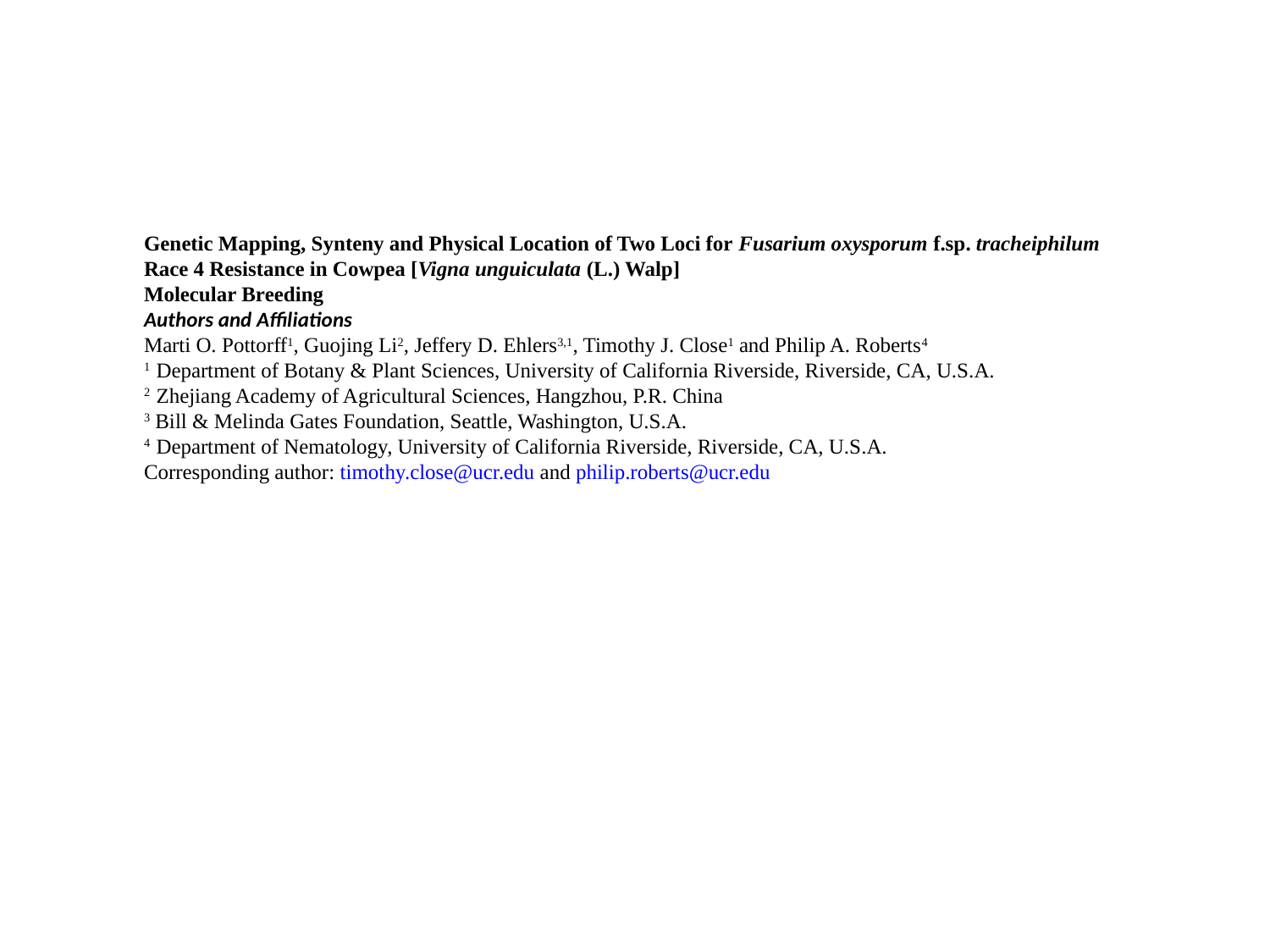

Genetic Mapping, Synteny and Physical Location of Two Loci for Fusarium oxysporum f.sp. tracheiphilum
Race 4 Resistance in Cowpea [Vigna unguiculata (L.) Walp]
Molecular Breeding
Authors and Affiliations
Marti O. Pottorff1, Guojing Li2, Jeffery D. Ehlers3,1, Timothy J. Close1 and Philip A. Roberts4
1 Department of Botany & Plant Sciences, University of California Riverside, Riverside, CA, U.S.A.
2 Zhejiang Academy of Agricultural Sciences, Hangzhou, P.R. China
3 Bill & Melinda Gates Foundation, Seattle, Washington, U.S.A.
4 Department of Nematology, University of California Riverside, Riverside, CA, U.S.A.
Corresponding author: timothy.close@ucr.edu and philip.roberts@ucr.edu

## Slide 2
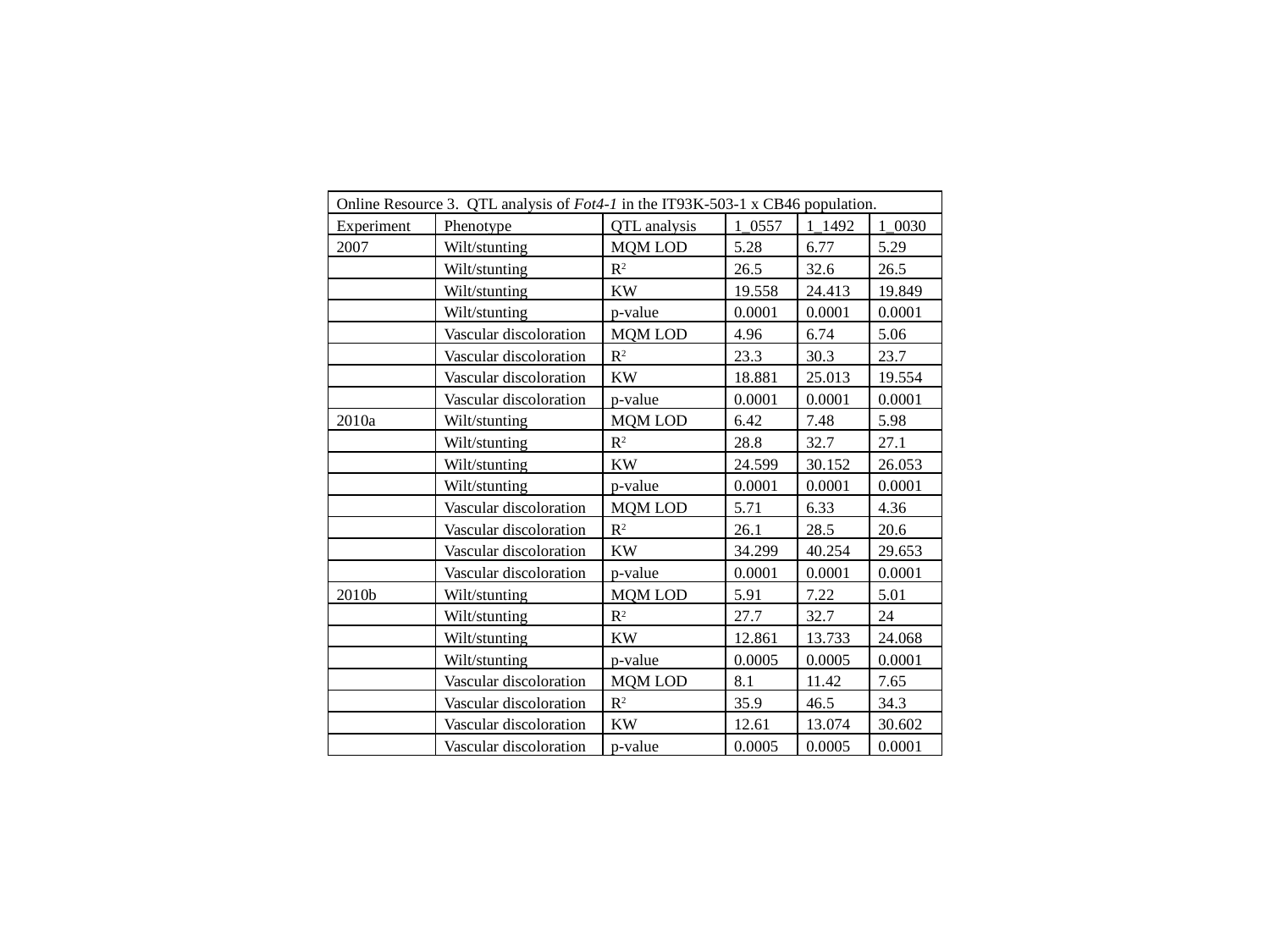

| Online Resource 3. QTL analysis of Fot4-1 in the IT93K-503-1 x CB46 population. | | | | | |
| --- | --- | --- | --- | --- | --- |
| Experiment | Phenotype | QTL analysis | 1\_0557 | 1\_1492 | 1\_0030 |
| 2007 | Wilt/stunting | MQM LOD | 5.28 | 6.77 | 5.29 |
| | Wilt/stunting | R2 | 26.5 | 32.6 | 26.5 |
| | Wilt/stunting | KW | 19.558 | 24.413 | 19.849 |
| | Wilt/stunting | p-value | 0.0001 | 0.0001 | 0.0001 |
| | Vascular discoloration | MQM LOD | 4.96 | 6.74 | 5.06 |
| | Vascular discoloration | R2 | 23.3 | 30.3 | 23.7 |
| | Vascular discoloration | KW | 18.881 | 25.013 | 19.554 |
| | Vascular discoloration | p-value | 0.0001 | 0.0001 | 0.0001 |
| 2010a | Wilt/stunting | MQM LOD | 6.42 | 7.48 | 5.98 |
| | Wilt/stunting | R2 | 28.8 | 32.7 | 27.1 |
| | Wilt/stunting | KW | 24.599 | 30.152 | 26.053 |
| | Wilt/stunting | p-value | 0.0001 | 0.0001 | 0.0001 |
| | Vascular discoloration | MQM LOD | 5.71 | 6.33 | 4.36 |
| | Vascular discoloration | R2 | 26.1 | 28.5 | 20.6 |
| | Vascular discoloration | KW | 34.299 | 40.254 | 29.653 |
| | Vascular discoloration | p-value | 0.0001 | 0.0001 | 0.0001 |
| 2010b | Wilt/stunting | MQM LOD | 5.91 | 7.22 | 5.01 |
| | Wilt/stunting | R2 | 27.7 | 32.7 | 24 |
| | Wilt/stunting | KW | 12.861 | 13.733 | 24.068 |
| | Wilt/stunting | p-value | 0.0005 | 0.0005 | 0.0001 |
| | Vascular discoloration | MQM LOD | 8.1 | 11.42 | 7.65 |
| | Vascular discoloration | R2 | 35.9 | 46.5 | 34.3 |
| | Vascular discoloration | KW | 12.61 | 13.074 | 30.602 |
| | Vascular discoloration | p-value | 0.0005 | 0.0005 | 0.0001 |
