# Supplementary material for: Genetic mapping, synteny, and physical location of two loci for Fusarium oxysporum f. sp. tracheiphilum race 4 resistance in cowpea [Vignaunguiculata (L.) Walp]
Source: Mol Breed. 2013 Dec 13;33(4):779–91. doi: 10.1007/s11032-013-9991-0 (PMC3956937; doi:10.1007/s11032-013-9991-0)
Supplement: Supplementary file 7 — Online Resource 7 Fot4-2 locus in the CB27 x 24-125B-1 and CB27 x IT82E-18/Big Buff populations, the cowpea consensus genetic map and the cowpea physical map (PPTX 63 kb) [file 11032_2013_9991_MOESM7_ESM.pptx]

## Slide 1
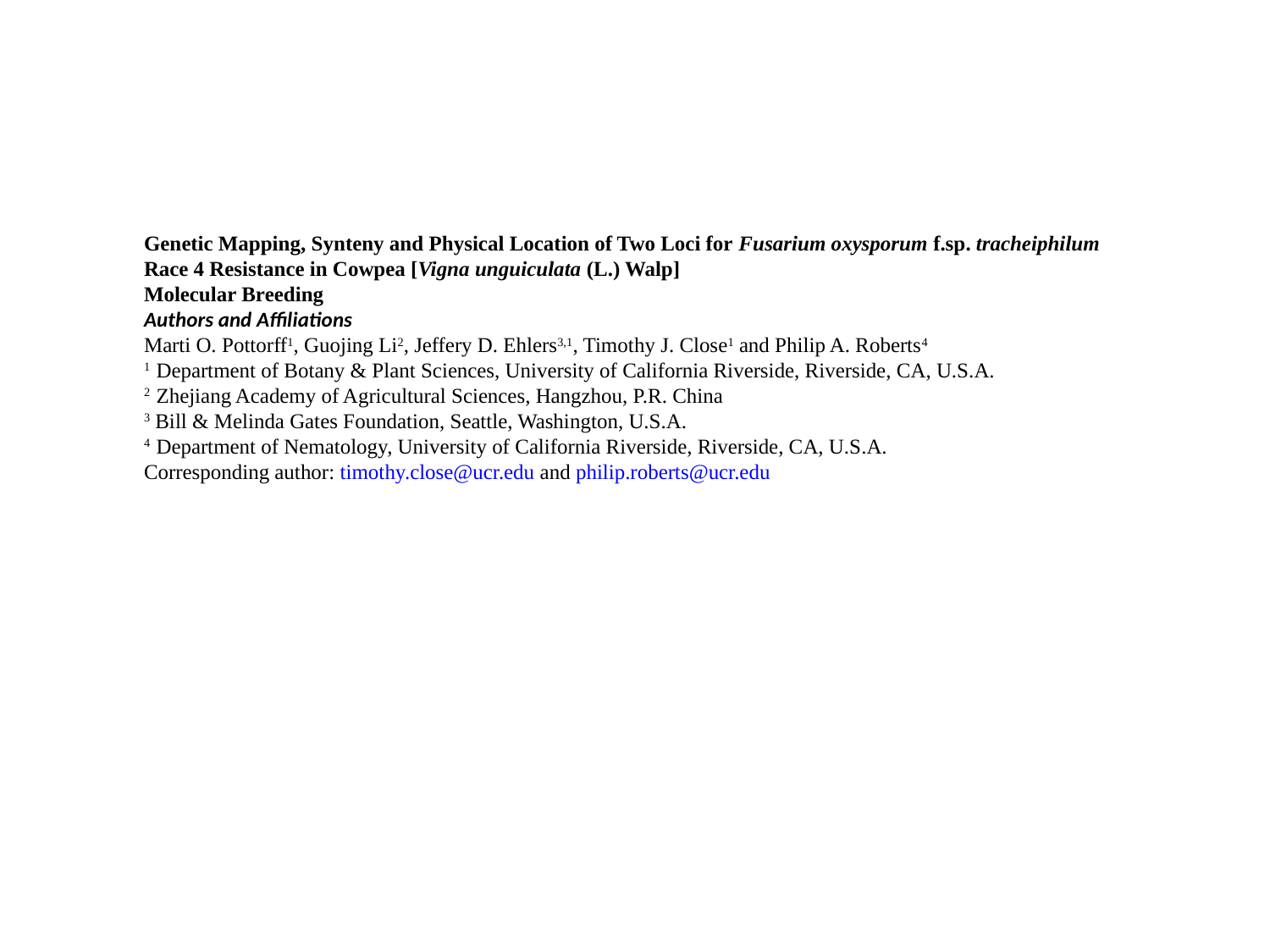

Genetic Mapping, Synteny and Physical Location of Two Loci for Fusarium oxysporum f.sp. tracheiphilum
Race 4 Resistance in Cowpea [Vigna unguiculata (L.) Walp]
Molecular Breeding
Authors and Affiliations
Marti O. Pottorff1, Guojing Li2, Jeffery D. Ehlers3,1, Timothy J. Close1 and Philip A. Roberts4
1 Department of Botany & Plant Sciences, University of California Riverside, Riverside, CA, U.S.A.
2 Zhejiang Academy of Agricultural Sciences, Hangzhou, P.R. China
3 Bill & Melinda Gates Foundation, Seattle, Washington, U.S.A.
4 Department of Nematology, University of California Riverside, Riverside, CA, U.S.A.
Corresponding author: timothy.close@ucr.edu and philip.roberts@ucr.edu

## Slide 2
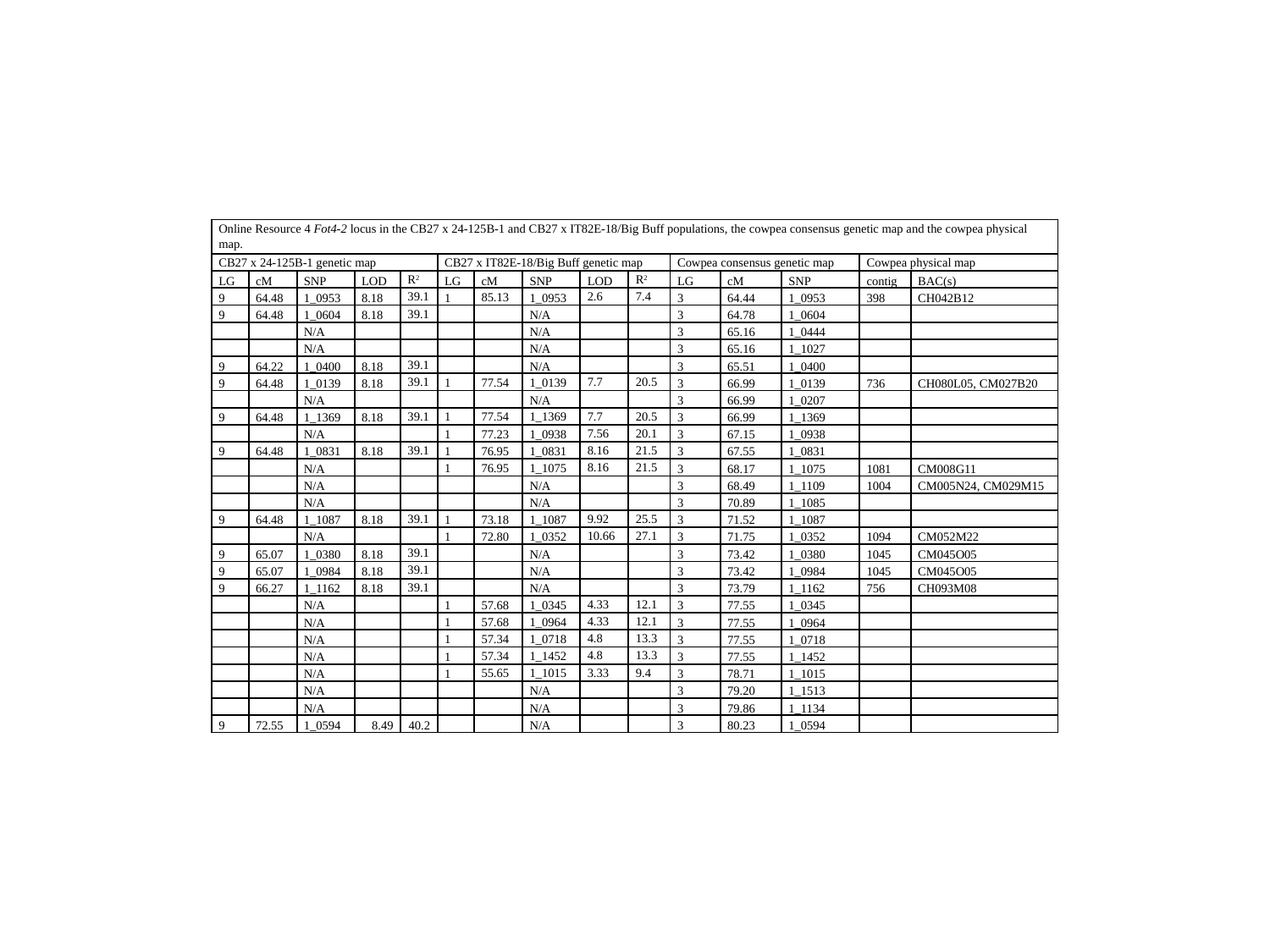

| Online Resource 4 Fot4-2 locus in the CB27 x 24-125B-1 and CB27 x IT82E-18/Big Buff populations, the cowpea consensus genetic map and the cowpea physical map. | | | | | | | | | | | | | | |
| --- | --- | --- | --- | --- | --- | --- | --- | --- | --- | --- | --- | --- | --- | --- |
| CB27 x 24-125B-1 genetic map | | | | | CB27 x IT82E-18/Big Buff genetic map | | | | | Cowpea consensus genetic map | | | Cowpea physical map | |
| LG | cM | SNP | LOD | R2 | LG | cM | SNP | LOD | R2 | LG | cM | SNP | contig | BAC(s) |
| 9 | 64.48 | 1\_0953 | 8.18 | 39.1 | 1 | 85.13 | 1\_0953 | 2.6 | 7.4 | 3 | 64.44 | 1\_0953 | 398 | CH042B12 |
| 9 | 64.48 | 1\_0604 | 8.18 | 39.1 | | | N/A | | | 3 | 64.78 | 1\_0604 | | |
| | | N/A | | | | | N/A | | | 3 | 65.16 | 1\_0444 | | |
| | | N/A | | | | | N/A | | | 3 | 65.16 | 1\_1027 | | |
| 9 | 64.22 | 1\_0400 | 8.18 | 39.1 | | | N/A | | | 3 | 65.51 | 1\_0400 | | |
| 9 | 64.48 | 1\_0139 | 8.18 | 39.1 | 1 | 77.54 | 1\_0139 | 7.7 | 20.5 | 3 | 66.99 | 1\_0139 | 736 | CH080L05, CM027B20 |
| | | N/A | | | | | N/A | | | 3 | 66.99 | 1\_0207 | | |
| 9 | 64.48 | 1\_1369 | 8.18 | 39.1 | 1 | 77.54 | 1\_1369 | 7.7 | 20.5 | 3 | 66.99 | 1\_1369 | | |
| | | N/A | | | 1 | 77.23 | 1\_0938 | 7.56 | 20.1 | 3 | 67.15 | 1\_0938 | | |
| 9 | 64.48 | 1\_0831 | 8.18 | 39.1 | 1 | 76.95 | 1\_0831 | 8.16 | 21.5 | 3 | 67.55 | 1\_0831 | | |
| | | N/A | | | 1 | 76.95 | 1\_1075 | 8.16 | 21.5 | 3 | 68.17 | 1\_1075 | 1081 | CM008G11 |
| | | N/A | | | | | N/A | | | 3 | 68.49 | 1\_1109 | 1004 | CM005N24, CM029M15 |
| | | N/A | | | | | N/A | | | 3 | 70.89 | 1\_1085 | | |
| 9 | 64.48 | 1\_1087 | 8.18 | 39.1 | 1 | 73.18 | 1\_1087 | 9.92 | 25.5 | 3 | 71.52 | 1\_1087 | | |
| | | N/A | | | 1 | 72.80 | 1\_0352 | 10.66 | 27.1 | 3 | 71.75 | 1\_0352 | 1094 | CM052M22 |
| 9 | 65.07 | 1\_0380 | 8.18 | 39.1 | | | N/A | | | 3 | 73.42 | 1\_0380 | 1045 | CM045O05 |
| 9 | 65.07 | 1\_0984 | 8.18 | 39.1 | | | N/A | | | 3 | 73.42 | 1\_0984 | 1045 | CM045O05 |
| 9 | 66.27 | 1\_1162 | 8.18 | 39.1 | | | N/A | | | 3 | 73.79 | 1\_1162 | 756 | CH093M08 |
| | | N/A | | | 1 | 57.68 | 1\_0345 | 4.33 | 12.1 | 3 | 77.55 | 1\_0345 | | |
| | | N/A | | | 1 | 57.68 | 1\_0964 | 4.33 | 12.1 | 3 | 77.55 | 1\_0964 | | |
| | | N/A | | | 1 | 57.34 | 1\_0718 | 4.8 | 13.3 | 3 | 77.55 | 1\_0718 | | |
| | | N/A | | | 1 | 57.34 | 1\_1452 | 4.8 | 13.3 | 3 | 77.55 | 1\_1452 | | |
| | | N/A | | | 1 | 55.65 | 1\_1015 | 3.33 | 9.4 | 3 | 78.71 | 1\_1015 | | |
| | | N/A | | | | | N/A | | | 3 | 79.20 | 1\_1513 | | |
| | | N/A | | | | | N/A | | | 3 | 79.86 | 1\_1134 | | |
| 9 | 72.55 | 1\_0594 | 8.49 | 40.2 | | | N/A | | | 3 | 80.23 | 1\_0594 | | |
