# Supplementary material for: Genetic mapping, synteny, and physical location of two loci for Fusarium oxysporum f. sp. tracheiphilum race 4 resistance in cowpea [Vignaunguiculata (L.) Walp]
Source: Mol Breed. 2013 Dec 13;33(4):779–91. doi: 10.1007/s11032-013-9991-0 (PMC3956937; doi:10.1007/s11032-013-9991-0)
Supplement: Supplementary file 8 — Online Resource 8 QTL analysis of Fot4-2 in the CB27 x 24-125B-1 population (PPTX 58 kb) [file 11032_2013_9991_MOESM8_ESM.pptx]

## Slide 1
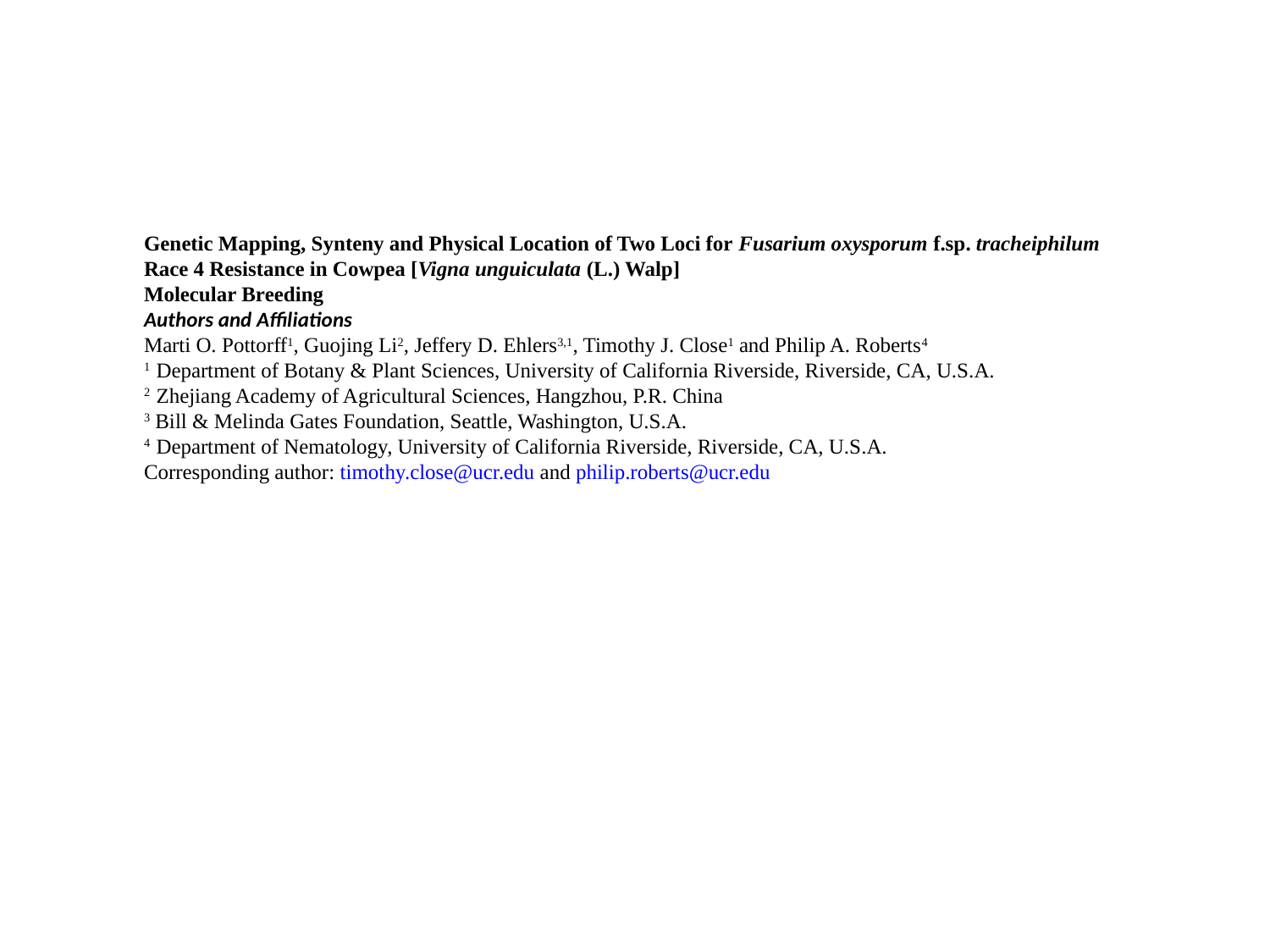

Genetic Mapping, Synteny and Physical Location of Two Loci for Fusarium oxysporum f.sp. tracheiphilum
Race 4 Resistance in Cowpea [Vigna unguiculata (L.) Walp]
Molecular Breeding
Authors and Affiliations
Marti O. Pottorff1, Guojing Li2, Jeffery D. Ehlers3,1, Timothy J. Close1 and Philip A. Roberts4
1 Department of Botany & Plant Sciences, University of California Riverside, Riverside, CA, U.S.A.
2 Zhejiang Academy of Agricultural Sciences, Hangzhou, P.R. China
3 Bill & Melinda Gates Foundation, Seattle, Washington, U.S.A.
4 Department of Nematology, University of California Riverside, Riverside, CA, U.S.A.
Corresponding author: timothy.close@ucr.edu and philip.roberts@ucr.edu

## Slide 2
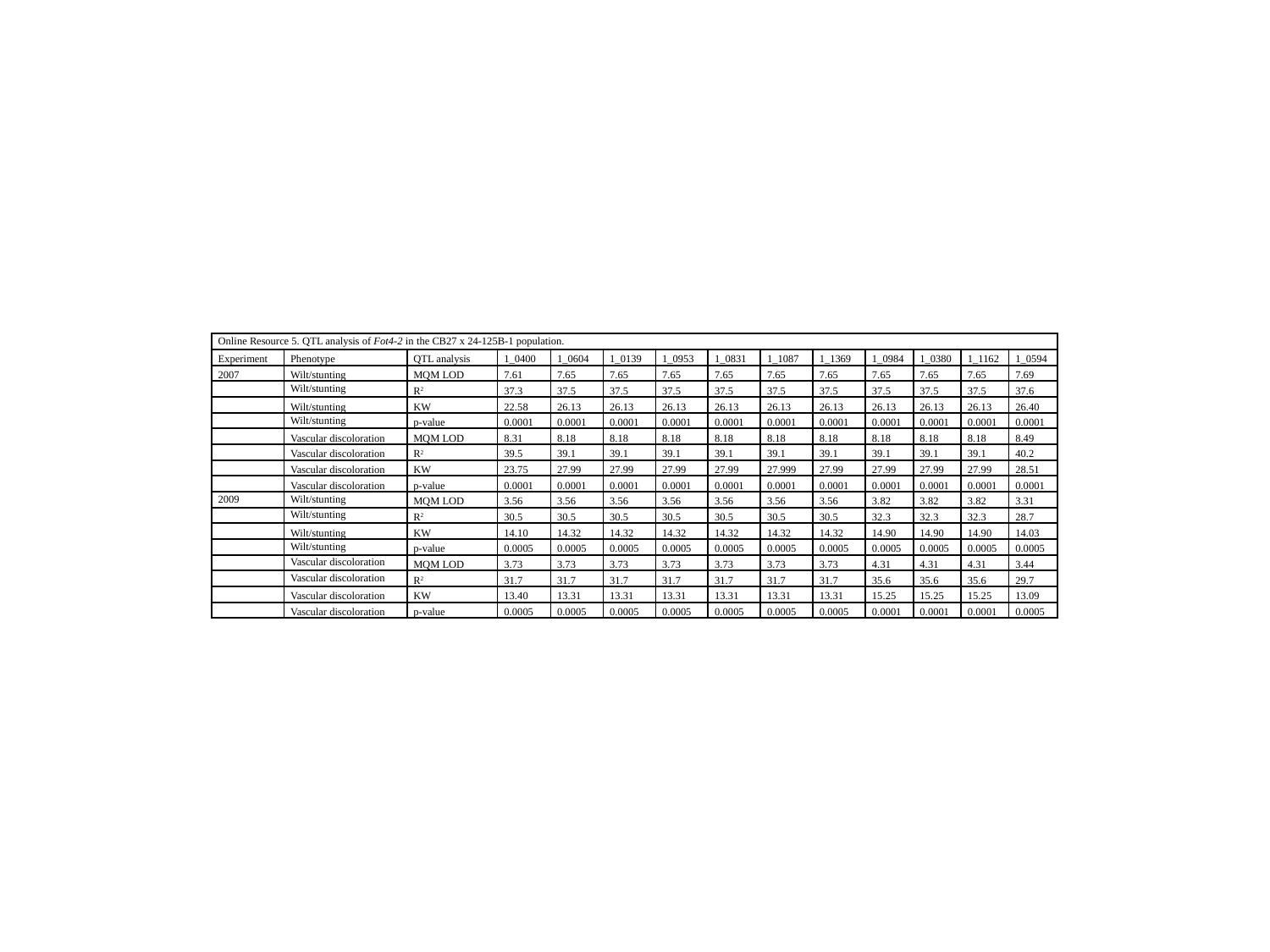

| Online Resource 5. QTL analysis of Fot4-2 in the CB27 x 24-125B-1 population. | | | | | | | | | | | | | |
| --- | --- | --- | --- | --- | --- | --- | --- | --- | --- | --- | --- | --- | --- |
| Experiment | Phenotype | QTL analysis | 1\_0400 | 1\_0604 | 1\_0139 | 1\_0953 | 1\_0831 | 1\_1087 | 1\_1369 | 1\_0984 | 1\_0380 | 1\_1162 | 1\_0594 |
| 2007 | Wilt/stunting | MQM LOD | 7.61 | 7.65 | 7.65 | 7.65 | 7.65 | 7.65 | 7.65 | 7.65 | 7.65 | 7.65 | 7.69 |
| | Wilt/stunting | R2 | 37.3 | 37.5 | 37.5 | 37.5 | 37.5 | 37.5 | 37.5 | 37.5 | 37.5 | 37.5 | 37.6 |
| | Wilt/stunting | KW | 22.58 | 26.13 | 26.13 | 26.13 | 26.13 | 26.13 | 26.13 | 26.13 | 26.13 | 26.13 | 26.40 |
| | Wilt/stunting | p-value | 0.0001 | 0.0001 | 0.0001 | 0.0001 | 0.0001 | 0.0001 | 0.0001 | 0.0001 | 0.0001 | 0.0001 | 0.0001 |
| | Vascular discoloration | MQM LOD | 8.31 | 8.18 | 8.18 | 8.18 | 8.18 | 8.18 | 8.18 | 8.18 | 8.18 | 8.18 | 8.49 |
| | Vascular discoloration | R2 | 39.5 | 39.1 | 39.1 | 39.1 | 39.1 | 39.1 | 39.1 | 39.1 | 39.1 | 39.1 | 40.2 |
| | Vascular discoloration | KW | 23.75 | 27.99 | 27.99 | 27.99 | 27.99 | 27.999 | 27.99 | 27.99 | 27.99 | 27.99 | 28.51 |
| | Vascular discoloration | p-value | 0.0001 | 0.0001 | 0.0001 | 0.0001 | 0.0001 | 0.0001 | 0.0001 | 0.0001 | 0.0001 | 0.0001 | 0.0001 |
| 2009 | Wilt/stunting | MQM LOD | 3.56 | 3.56 | 3.56 | 3.56 | 3.56 | 3.56 | 3.56 | 3.82 | 3.82 | 3.82 | 3.31 |
| | Wilt/stunting | R2 | 30.5 | 30.5 | 30.5 | 30.5 | 30.5 | 30.5 | 30.5 | 32.3 | 32.3 | 32.3 | 28.7 |
| | Wilt/stunting | KW | 14.10 | 14.32 | 14.32 | 14.32 | 14.32 | 14.32 | 14.32 | 14.90 | 14.90 | 14.90 | 14.03 |
| | Wilt/stunting | p-value | 0.0005 | 0.0005 | 0.0005 | 0.0005 | 0.0005 | 0.0005 | 0.0005 | 0.0005 | 0.0005 | 0.0005 | 0.0005 |
| | Vascular discoloration | MQM LOD | 3.73 | 3.73 | 3.73 | 3.73 | 3.73 | 3.73 | 3.73 | 4.31 | 4.31 | 4.31 | 3.44 |
| | Vascular discoloration | R2 | 31.7 | 31.7 | 31.7 | 31.7 | 31.7 | 31.7 | 31.7 | 35.6 | 35.6 | 35.6 | 29.7 |
| | Vascular discoloration | KW | 13.40 | 13.31 | 13.31 | 13.31 | 13.31 | 13.31 | 13.31 | 15.25 | 15.25 | 15.25 | 13.09 |
| | Vascular discoloration | p-value | 0.0005 | 0.0005 | 0.0005 | 0.0005 | 0.0005 | 0.0005 | 0.0005 | 0.0001 | 0.0001 | 0.0001 | 0.0005 |
