# Supplementary material for: Genetic mapping, synteny, and physical location of two loci for Fusarium oxysporum f. sp. tracheiphilum race 4 resistance in cowpea [Vignaunguiculata (L.) Walp]
Source: Mol Breed. 2013 Dec 13;33(4):779–91. doi: 10.1007/s11032-013-9991-0 (PMC3956937; doi:10.1007/s11032-013-9991-0)
Supplement: Supplementary file 9 — Online Resource 9 QTL analysis of Fot4-2 in the CB27 x IT82E-18 /Big Buff population (PPTX 54 kb) [file 11032_2013_9991_MOESM9_ESM.pptx]

## Slide 1
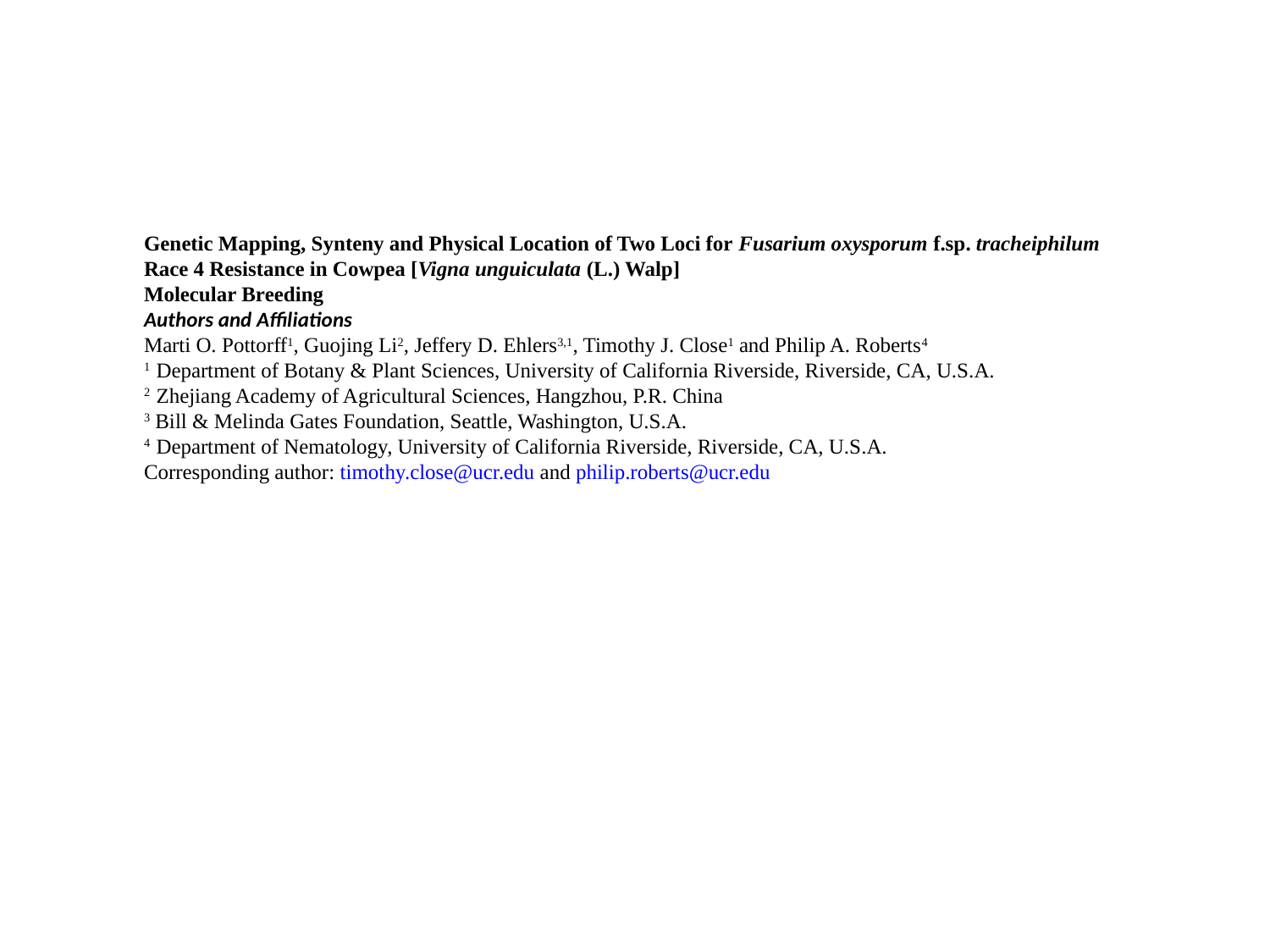

Genetic Mapping, Synteny and Physical Location of Two Loci for Fusarium oxysporum f.sp. tracheiphilum
Race 4 Resistance in Cowpea [Vigna unguiculata (L.) Walp]
Molecular Breeding
Authors and Affiliations
Marti O. Pottorff1, Guojing Li2, Jeffery D. Ehlers3,1, Timothy J. Close1 and Philip A. Roberts4
1 Department of Botany & Plant Sciences, University of California Riverside, Riverside, CA, U.S.A.
2 Zhejiang Academy of Agricultural Sciences, Hangzhou, P.R. China
3 Bill & Melinda Gates Foundation, Seattle, Washington, U.S.A.
4 Department of Nematology, University of California Riverside, Riverside, CA, U.S.A.
Corresponding author: timothy.close@ucr.edu and philip.roberts@ucr.edu

## Slide 2
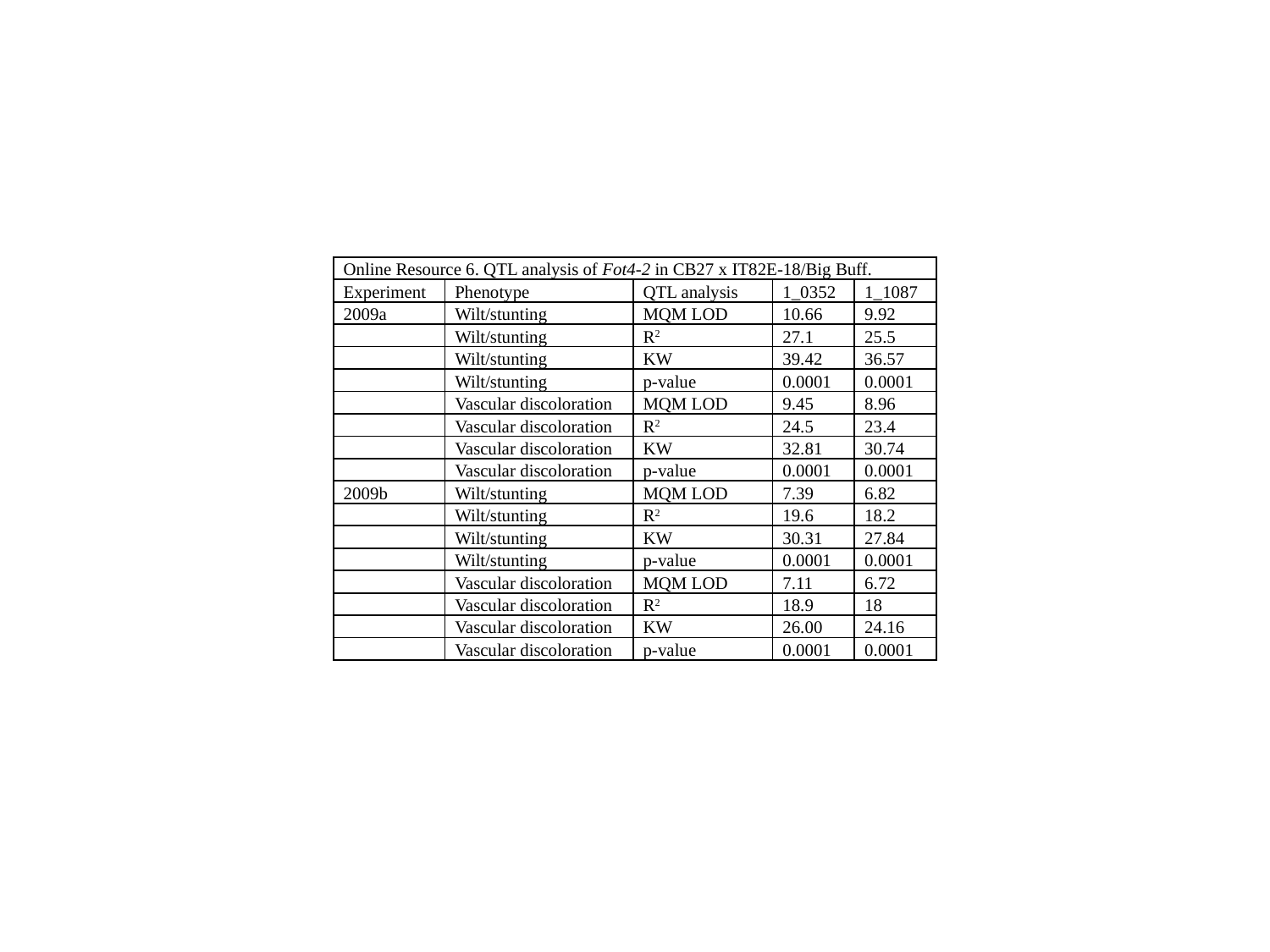

| Online Resource 6. QTL analysis of Fot4-2 in CB27 x IT82E-18/Big Buff. | | | | |
| --- | --- | --- | --- | --- |
| Experiment | Phenotype | QTL analysis | 1\_0352 | 1\_1087 |
| 2009a | Wilt/stunting | MQM LOD | 10.66 | 9.92 |
| | Wilt/stunting | R2 | 27.1 | 25.5 |
| | Wilt/stunting | KW | 39.42 | 36.57 |
| | Wilt/stunting | p-value | 0.0001 | 0.0001 |
| | Vascular discoloration | MQM LOD | 9.45 | 8.96 |
| | Vascular discoloration | R2 | 24.5 | 23.4 |
| | Vascular discoloration | KW | 32.81 | 30.74 |
| | Vascular discoloration | p-value | 0.0001 | 0.0001 |
| 2009b | Wilt/stunting | MQM LOD | 7.39 | 6.82 |
| | Wilt/stunting | R2 | 19.6 | 18.2 |
| | Wilt/stunting | KW | 30.31 | 27.84 |
| | Wilt/stunting | p-value | 0.0001 | 0.0001 |
| | Vascular discoloration | MQM LOD | 7.11 | 6.72 |
| | Vascular discoloration | R2 | 18.9 | 18 |
| | Vascular discoloration | KW | 26.00 | 24.16 |
| | Vascular discoloration | p-value | 0.0001 | 0.0001 |
